# Mesenchymal stem cells in cardiac regeneration: a detailed progress report of the last 6 years (2010–2015)

**DOI:** 10.1186/s13287-016-0341-0

**Published:** 2016-06-04

**Authors:** Aastha Singh, Abhishek Singh, Dwaipayan Sen

**Affiliations:** 1 School of Bio Sciences and Technology, VIT University, Vellore, India; 2 Cellular and Molecular Therapeutics Laboratory, Centre for Biomaterials, Cellular and Molecular Theranostics (CBCMT), VIT University, Vellore, 632014 Tamil Nadu India

**Keywords:** Mesenchymal stem cells, Cardiac regeneration, Niche hypothesis, Cell therapy, Cell transplantation

## Abstract

Mesenchymal stem cells have been used for cardiovascular regenerative therapy for decades. These cells have been established as one of the potential therapeutic agents, following several tests in animal models and clinical trials. In the process, various sources of mesenchymal stem cells have been identified which help in cardiac regeneration by either revitalizing the cardiac stem cells or revascularizing the arteries and veins of the heart. Although mesenchymal cell therapy has achieved considerable admiration, some challenges still remain that need to be overcome in order to establish it as a successful technique. This in-depth review is an attempt to summarize the major sources of mesenchymal stem cells involved in myocardial regeneration, the significant mechanisms involved in the process with a focus on studies (human and animal) conducted in the last 6 years and the challenges that remain to be addressed.

**Electronic supplementary material:**

The online version of this article (doi:10.1186/s13287-016-0341-0) contains supplementary material, which is available to authorized users.

## Background

Stem cells are capable of differentiating into cells of the same type, which in turn give rise to other kinds of cells [1]. Stem cells can be classified on the basis of their origin and potential to differentiate. Based on origin, these cells are of two types: embryonic stem cells (ESCs) and non-ESCs. The non-ESCs are present in two forms: haematopoietic stem cells (HSCs) that differentiate into different blood cells and are CD34^+^; and the less differentiated mesenchymal stem cells (MSCs). Under the second classification system, stem cells can be categorized as totipotent, pluripotent and multipotent, based on their potential to differentiate into different cell types. All stem cells have three common features, namely boundless self-renewal capacity, potential for asymmetric divisions and an irreversible differentiation process [2].

Cardiovascular diseases account for the highest mortality in the western countries of the world [3]. Unlike lower vertebrates like zebrafish [4], adult mammals do not possess the capacity for natural heart regeneration throughout their lifetime [5] and hence several therapeutic measures have been investigated for myocardial regeneration and repair. Out of these numerous approaches, the first clinical trials about a decade ago bolstered stem cell therapy as one of the potential strategies utilized in the cure of these disorders. The current research in the field of cardiac regenerative medicine thus attempts to stimulate the endogenous regenerative mechanisms via cell therapy for conditions such as myocardial infarction (MI). This is achieved by intermingling of two components: a cardiomyocyte source as the target for regeneration; and a non-myocardial tissue acting as a source for regeneration in an effective cardiac environment [5].

This review focuses on summarizing all studies concerning MSCs in terms of in-vivo and clinical observations in the last 6 years (2010–2015), following a critical evaluation of its cardiomyogenic potential as well as the clinical trials.

## Main text

### Importance of the MSC niche for cardiac regeneration

The Niche hypothesis [6] proposes the existence of an optimal microenvironment for stem cells. This concept has been pledged to explain the hierarchy of stem cells, with different degrees of differentiation capacity [2].

In 2011, Vunjak-Novakovic and Scadden [7] categorized the cellular and acellular components into key factors such as regulatory molecules (cytokines, O_2_, nutrients), extracellular matrix (ECM) (structure, stiffness, immobilized and released factors), other cells (cell–cell contact, paracrine and autocrine signals) and physical factors (stretch, electrical signals). Many studies have concentrated on the hypoxic environment of the MSC niche [8]. Since oxygen tension (i.e. O_2_ levels below 8–9 %) [9] can lead to cellular damage and apoptosis, hypoxia preconditioning of MSCs and pro-survival gene overexpression (e.g. *Akt* gene) can lead to reduction in hypoxia-induced cell death [10]. Hypoxia stimulation can be attained by transducing hypoxia-inducible factor *(HIF)-1α* [11] lentivirus vector into the MSCs, which increases proliferation and differentiation rates of the mesenchymal lineages. Cellular repressor of E1A-stimulated genes (*CREG*) also plays a role in activating *HIF-1α*, but not *HIF-1β*, by degrading a key protein that degrades *HIF-1α* [12]. This in turn modulates the paracrine signalling, resulting in upregulation of angiogenic factors such as vascular endothelial growth factor (*VEGF*) [13, 14], stromal cell-derived factor-1α (*SDF-1α*) [14], hepatocyte growth factor (*HGF*) [15] and *IL-6* [10]. *CREG* also leads to reduction in fibrotic tissue and cardiomyocyte proliferation [11]. MSCs have also been studied to release extracellular vesicles under hypoxic conditions, resulting in neoangiogenesis and enhanced cardiac functioning [16]. Human tissue kallikrein (*TK*) gene [17], trimetazidine (*TMZ*) [18] and midkine [19], when transduced or overexpressed in MSCs and transplanted into rat hearts, were found to provide more resistance to hypoxia-induced apoptosis, inflammatory damage and cardiac injury. Overall the MSCS promoted enhanced neovascularization and cardiac functional recovery. TK-MSCs have also been shown to exhibit enhanced *VEGF* expression and reduced *caspase-3* activity [17], while *TMZ* preconditioning of MSCs led to increased levels of the anti-apoptotic protein *Bcl-2* [20]. However, *TMZ *has been observed to induce adverse drug reactions associated with Parkinson’s syndrome [21] and thus requires careful evaluation before being established as a promising therapeutic agent. *Let7b*-transfected MSCs also target the *caspase-3* expression for upregulating the pro-survival genes such as *p-ERK*, *Bcl-2* and *p-MEK* and result in improved left ventricular ejection fraction (LVEF) in the rat MI model [22].

### Adult stem cells in regenerative medicine

#### Adult stem cells

Adult stem cells were thought to have a multipotent lineage, but recent research has highlighted their pluripotent nature, transdifferentiating into various progenies [23]. The progenies in turn form cells of multipotent lineages, such as HSCs and MSCs [24]. HSCs are pluripotent cells that further differentiate into blood cells of lymphoid (B, T and NK cells) and myeloid (monocyte, granulocyte, megakaryocyte and erythrocyte) lineages [25]. They are therefore mainly involved in haematopoiesis and treatment of related diseases. MSCs have shown promising regenerative abilities in stimulating cardiomyocyte formation, in association with a Notch ligand, Jagged 1 [26]. MSCs along with other pluripotent stem cells have been said to be an effective tool for angiogenesis, cardiac regeneration and hence cardiac tissue revitalization [27], and they have also been established to be more effective than HSCs for treatment of MI in nude rat model [28].

**Fig. 1:**
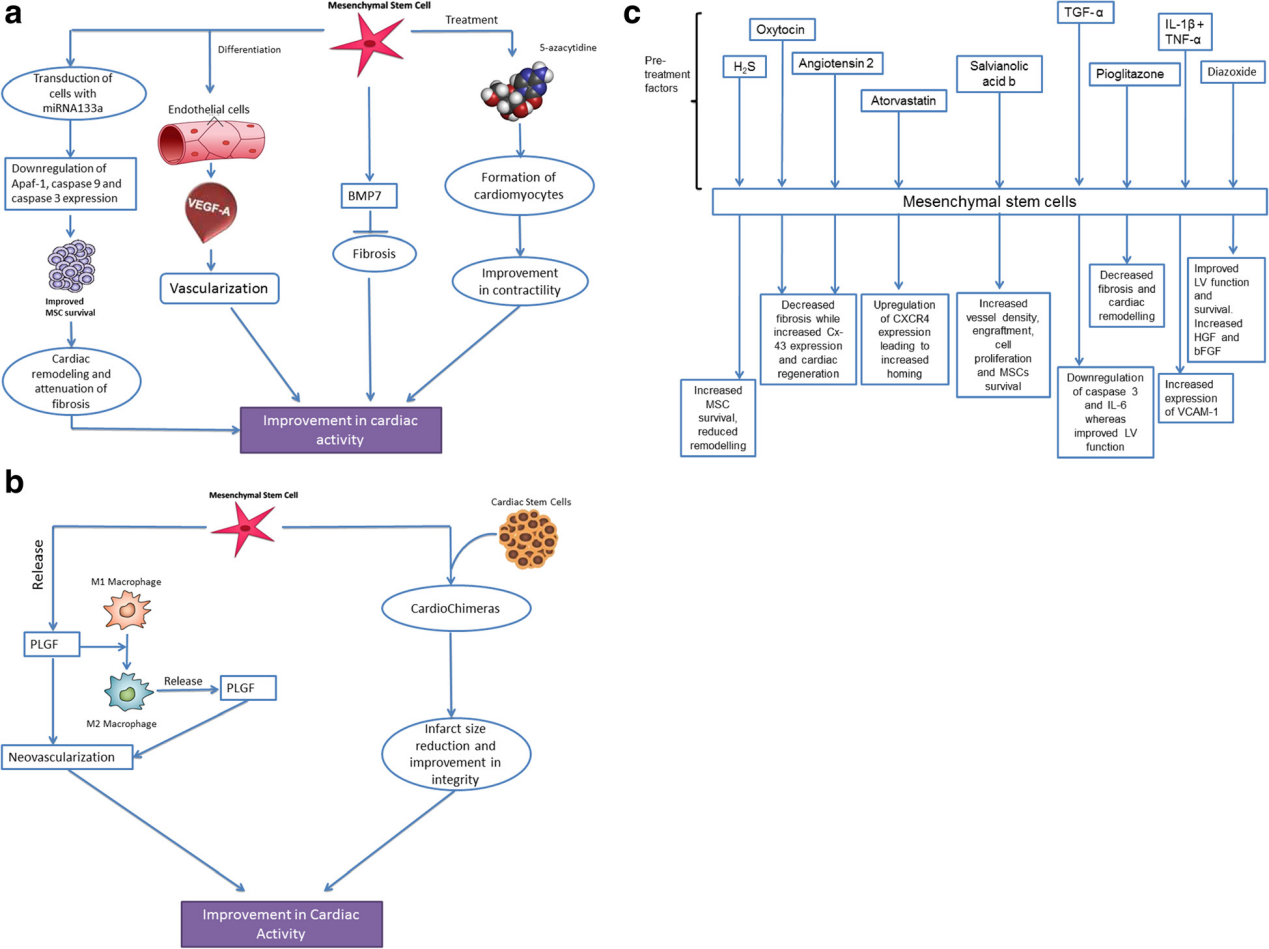
Mechanisms of action of MSCs for cardiac regeneration. (**a**) *miR-133a* downregulates the expression of* Apaf-1* and *caspase 3* and *9*, leading to attenuated fibrosis. ECs producing growth factors such as *VEGF-A* help in recruiting the peripheral stem cells, along with coordinating the differentiation of MSCs into endothelial cells, thereby leading to vascularization. *BMP7* expressed by MSCs lead to inhibition of fibrosis on counteraction of *TGF-β* secreted by macrophages. 5-azacytidine induces differentiation of MSCs into cardiomyocyte, thereby mitigating cardiac contractibility. (**b**) *PLGF*-induced macrophage polarization from M1 to M2 promotes neovascularization. CardioChimeras are mono-nucleate fusion of CSCs and MSCs which have exclusive growth kinetics, and have proven to be superior to the parent precursors. (**c**) MSCs pretreated with various compounds show cryoprotective effects along with enhanced cardiomyogenesis and improved heart function.  *bFGF* basic fibroblast growth factor, *CSC *cardiac stem cell,* EC *endothelial cell, *HGF* hepatocyte growth factor, *LV* left ventricular, *MSC* mesenchymal stem cell, *PLGF* platelet-derived growth factor, *TGF* tumor growth factor, *VCAM* vascular cell adhesion molecule, *VEGF* vascular endothelial growth factor

Cardiac stem cells (CSCs) are multipotent in nature, and are capable of differentiating into vascular cells and cardiomyocytes [29]. These can be differentiated from hMSCs on the basis of their inability to differentiate into osteocytes and adipocytes [30]. The presence of *c-kit* marker is used as an interpretation for cardiac progenitor cells (CPCs) [31]. The cardiac regenerative capacity of CSCs was studied against that of MSCs and enhanced levels of histone acetylation at the promoter regions of the cardiac specific genes were found to be higher in CSCs than in MSCs [32]. This observation indicates that CSCs have a higher potential to differentiate into cardiomyocytes than MSCs and has further been supported by animal studies showing higher modulatory characteristics of CSCs, such as reduced scar size and vascular overload [33, 34]. Fetal cardiac MSCs (fC-MSCs) are said to be primitive stem cell types with the ability to differentiate into osteocytes, adipocytes, neuronal cells and hepatocytic cells [35]. These cells demonstrate a high degree of plasticity and have a wide spectrum of therapeutic applications. Cardiac colony-forming unit fibroblasts (CFU-Fs) are another population of cells which are pro-epicardium derived and resemble MSCs. According to a study by Williams et al. [36], combination of hCSCs and hMSCs enhance the therapeutic response by producing greater infarct size reduction post MI. Yet another study highlighted the prospect of cardiac CFU-Fs holding higher therapeutic potential than bone marrow-derived MSCs (BM-MSCs) for cardiac repair [37]. The formation of CFU-Fs has been said to be enhanced by treatment of BM-MSCs with 1,25-dihydroxy vitamin D_3_ [38]. Adult stem cells tend to undergo cardiomyogenesis due to stimulation by oxytocin [39] (Fig. 1c) and paracrine factors released by human cardiac explants which leads to expression of cardiac-specific markers and differentiation of the MSCs into cardiomyocyte-like cells [40]. In a study conducted to estimate the efficacies of different stem cells, the results suggested that unrestricted somatic stem cells are more effective in providing cardiac functionality to the damaged tissue post MI than the BM-MSCs, even though their capacity to repair the damage is moderate [41]. Another interesting subfamily of the CSCs is cardiac resident stem cells (CRSCs) which can be obtained from adult human atrial appendages. These stem cells when administered with W8B2 antigen exhibit cardiogenic differentiation capacity, along with secretion of a variety of angiogenic, inflammatory, chemotaxic and cell growth and survival cytokines [42].

### MSCs: a promising source of cell-based therapy

#### General characteristics

Mesenchymal cells, being multipotent stem cells, can differentiate into several cell types such as mesodermal lineage cells (adipocyte, osteoblast, chondrocyte) [43] and myogenic lineage [44]. This feature of the MSC makes it an alluring therapeutic agent. According to the Tissue Stem Cell Committee of the International Society of Cellular Therapy [45], the basic criteria to categorize stem cell as MSC include following three key features:

- The cells must be plastic adherent under basic culture conditions.
- The cells should express CD73, CD90 and CD105, lacking the expression of CD11b, CD19 or CD79α, CD14, CD34, CD45 and HLA-DR surface molecules.
- The cells must be able to differentiate to adipocytes, chondrocytes and osteoblasts in vitro.

MSCs are said to exhibit immunomodulatory effects by virtue of their inhibitory effect towards both B-cell and T-cell proliferation [46], along with dendritic and NK cells, to promote allograft survival. In contrast, some studies have suggested the immunogenicity of MSCs, leading to proliferation of T cells towards infused MSCs and rejection of skin allografts by engendering functional memory T cells [47]. A very recent study has established the characteristics of human MSCs to be phenotypically and physiologically similar to human cardiac myofibroblasts. This study was concluded based on the positive staining of hMSCs for *α-SMA*, *NMMIIB*, *ED-A* fibronectin, vimentin and *sp1D8* (collagen type I), which was similar to that of cardiac myofibroblasts [48].

### Cardiomyogenic potential of MSCs obtained from different sources

MSCs are present in almost all tissues of the body and are mainly located in the perivascular alcove [49]. These can be derived from disparate adult (e.g. peripheral blood, adipose tissue, bone marrow) and neonatal (umbilical cord, amnion, cord blood and placenta) tissues [49], based on their therapeutic application. Although bone marrow represents the major source of MSCs in the body, it does not qualify as a viable isolation source of the cells due to high-grade viral infection and a substantial reduction in the proliferative capacity of the cells with age [50]. Also, MSC extraction from bone marrow is an invasive procedure, which causes immense pain to the patients and can also cause an infection [49]. Thus, MSCs derived from peripheral blood [51], heart [29], lung [52] and adipose tissue [53] have been explored for their biological properties, differentiation capacities and surface marker expression. Also, the cells obtained from neonatal tissues have been found to have superior biological properties as compared with BM-MSCs due to their ready availability, use of non-invasive techniques and avoidance of ethical problems [49]. A study categorized BM-MSCs based on their surface differentiation antigens and found that SCA-1^+^/CD31^+^/CD45^+^ subgroups displayed substantial cardiac improvement capacity, as compared to other BM-MSC subgroups such as SCA-1^+^/CD45^–^/CD31^–^, SCA-1^+^/CD45^+^/CD31^–^ or SCA-1^+^/CD45^–^/CD31^+^ [54]. Several animal cell lines have also been established to be used as biological tools for ex-vivo expansion and MSC differentiation into a definite lineage. One such MSC cell line was obtained using a porcine model, which when treated with 5-aza differentiated into cells containing positive cardiac phenotypic markers such as connexin-43 (*Cx-43*) and α-actin [55]. A study using porcine model demonstrated the use of histological staining as a feasible method to study the effect of these MSCs in myocardial regeneration [56]. MSCs obtained from patients with either coronary artery disease (CAD) or diabetes mellitus (DM), or both, help ameliorate cardiac function on transplantation but diabetes in a patient reduces the myocardial protection and proliferative capacity in hMSCs, as compared with CAD [57]. *Bcl-2* is family of proteins having a critical role in regulating anti-apoptotic pathways and cell death inhibition [58]. This feature of higher protective and proliferative capacity has been attributed to the lower expression of *Bcl-2* in CAD + DM patients compared with the CAD-only group [57].

According to one study [59], the traditional therapy techniques have been effective in treatment of acute diseases and improving a patient’s lifespan, but they do not serve to provide a permanent cure, thereby leaving the patients with protracted disease. On the contrary, cardiovascular regenerative medicine prevents further disease advancement by replacing the damaged cells with cardiac myocytes obtained from stem cells [60]. This is possible because stem cells are responsible for the generation and maintenance of terminally differentiated cell populations in tissues that undergo continuous turnover [2]. For instance, a study conducted by Brunt et al. investigated the myogenic differentiation based on age, where bone marrow MSCs were obtained from cardiovascular patients and a protein evaluation was conducted to estimate the *β-catenin* nuclear translocation in these patients. The study concluded with a first-time discovery of increased *β-catenin* bioavailability leading to myogenic differentiation and the *WNT/β-catenin* network as a potential target for reinvigoration of MSCs [61]. Regenerative medicine has explored several options in order to establish the use of MSCs as an expedient and more pragmatic technique towards cardiac regeneration from the various possible sources of regenerative tissue.

#### Bone marrow

Differentiation of scar tissue into cardiomyocytes can be instigated by transplanting bone marrow cells into the tissue and thereby restoring the myocardial function [62]. BM-MSCs have shown promising potential in cardiac repair due to their powerful proliferative capacity [63, 64], their ability to reduce the infarct size [65] and their ability to change the milieu of the damaged cardiac tissue to upregulate *VEGF* [66]. These have also been studied specifically for differentiation of CSCs [67]. For the first time, Cai et al. [68] demonstrated the use of these MSCs for the treatment of isopreterenol-induced myocardial hypertrophy. Another interesting observation in this study included the significance of inhibition of *VEGF*, and not fibroblast growth factor (*FGF*) or insulin-like growth factor, which restricted the protective effects of BM-MSCs on the hypertrophic condition [68]. Having mentioned this, the combined effect of BM-MSCs along with basic fibroblast growth factor (*bFGF*)-binding ECM has been observed to improve the left ventricular (LV) function and enhance myocardial regeneration [69]. One of the most effective delivery methods for the treatment has been observed to be via the retrograde infusion of the two [70]. Mixed treatment of BM-MSCs with endothelial progenitor cells (EPCs) pre-treated with salvianolic acid B results in reduced infarct area and enhanced stem cell proliferation [71]. BM-MSCs can be tracked by labelling them with superparamagnetic iron oxide (SPIO) nanoparticles in any MI rat [72] or swine model [73] and locating the shortened T2 value on the MRI scan. Similarly, quantum dots have been recently identified as another medium to label and track the cells, both in vitro and in vivo [74]. Emmert et al. [75] aligned a series of methods of cell tracking and imaging, including micron-sized iron-oxide labelling (MPIO), MRI, micro-CT flow cytometry and PCR followed by immunohistochemstry, in intra-uterine and intramyocardial (i.m.) BM-MSC transplantation pre-immune sheep models. The multipotency of these cells has been confirmed by a study based on human MSCs, which led to their differentiation into adipocytes, chondrocytes and osteoblasts [48]. Despite the similarity of these cells with cardiac myofibroblasts, they remain different due to their proliferative and differentiation properties, which are characteristic of MSCs. The repair coordinated by BM-MSCs is mainly mediated by causing relief from heart failure symptoms, and improving blood flow to the myocytes [76]. Also, bone marrow was the first source identified for MSCs, but several alternatives are being explored due to the invasive and painful extraction process.

BM-MSCs have been studied to transdifferentiate into cardiomyocytes, which involves a negative regulation by histone deacetylase 1 (*HDAC1*) [77]. *HDAC1* when knocked down leads to directed differentiation of the MSCs into cardiac cells. Multipotent BM-MSCs when reprogrammed into pluripotent cells result in MSC-derived induced pluripotent stem cells (MiPS), which express cardiac-specific transcription factors and form spontaneously beating cardiac progenitors [78]. These MiPS-derived progenitors engender infarcted heart and lead to improvement in global heart function. Bone marrow MSC/silk fibroin/hyaluronic acid (BMSC/SH) was implanted into myocardial infarcted rat hearts, where the condition was obtained by cryo-injury technique [79]. In comparison with the control and the other experimental models, BMSC-SH proved to improve the thickness of the LV wall, reduce apoptosis, promote neovascularization and stimulate several paracrine factors (e.g. *VEGF*), thereby compiling the advantages of the bioactive SH patches and stem cell therapy. In another study, the BM-MSCs were transplanted with induced (iBM-MSC) and uninduced (uBM-MSC) BM-MSCs in MI-induced rat hearts. As per the results obtained, the iBM-MSC-treated hearts showed improved fractional shortening as compared with any of the other models. Thus, iBM-MSC implantation has been considered as another potential therapeutic strategy for post-infarcted heart failure [80].

A combined therapy of BM-MSCs with Tanshinone IIA (Tan IIA) increased the migratory rate of the cells to the ischaemic region by promoting *SDF-1α* expression in the area, which was suppressed by AMD3100 (a CXC chemokine receptor 4 blocker (*CXCR4*)) [81]. This finding indicated the role of *SDF1/CXCR4* in BM-MSC migration. SDF-1 recruits the MSCs from bone marrow through a *CXCR4*-dependent mechanism [82] and when transfected into MSCs results in improved viability of the cells in infarcted hearts, thereby preserving the contractile function along with improving the paracrine action of the cells [83]. Similarly, TG-0054, a *CXCR4* antagonist, was studied in debilitating MI and cardiac dysfunction after 12 weeks of the treatment. This functional improvement is attributed to the ability of TG-0054 to mobilize the CD271-MSCs and reduce both plasma and myocardial cytokine levels [84]. BM-MSCs overexpressing myocardin-related transcription factor-A (*MRTF-A*) prevent primary cardiomyocyte apoptosis caused by H_2_O_2_, and thus help in reversing the cardiac damage after MI [85]. Similarly, overexpression of *CREG* in intramyocardially implanted BM-MSCs resulted in increased angiogenesis and reduced apoptosis and fibrosis [12]. Also, BM-MSCs treated with 5-aza along with exposure to 2G-hypergravity, when transplanted into a rat MI model, showed positive cardiac markers such as *Nkx2.5*, *Mef-2* and *GATA-4* indicating cardiac differentiation and functional recovery [86]. When *GATA-4* and *Nkx2.5* are transfected into BM-MSCs which are then co-cultured in the myocardial environment, the differentiation capacity of the cells increases along with the reparative capacity [87]. Another study compared rat BM-MSCs transfused with 5-aza to those exposed to electrical stimulation [88]. The results obtained showed higher levels of *Cx-43* and *Mef-2c* in the second group as compared with the first. This instigated the idea of electrically-stimulated MSC differentiation into cardiomyocytes. Similar results were obtained when a recombinant cocktail consisting of *IL-6*, *FGF-2*, *α-thrombin*, *BMP-4*, *TGF-β1* [89], retinoic acid, activin-A and insulin-like growth factor was transduced into hMSCs in order to guide cardiopoiesis [90]. Bone marrow mononuclear cells (BM-MNC) are an attractive source of MSCs [91] due to the ease of extraction of the cells. Comparing both of these bone-marrow-derived populations, MSCs result in higher vascularization, smaller infarct size [92] and improved LVEF [93] with respect to mononuclear cells [94]. BM-MSCs have been shown to degrade functionally and quantitatively with increase in age of patients undergoing successful reperfusion treatment, and hence this aspect of the MSCs needs to be explored further [95]. Prostaglandin E_1_ protects BM-MSCs against serum-deprived induced apoptosis by decreasing *Bax* and *caspase-3* expression levels and increasing *Bcl-2* expression [96]. In one study, bone marrow cells derived from heart failure patients were shown to express higher levels of remodelling enzymes and pathways regulating tissue remodelling, scar formation and maturation. This was attributed the increase in CD146^+^/*SMA-α* myofibroblast frequency [97]. Beyond this, BM-MSCs have shown to promote *c-kit*^*+*^ CSC differentiation via the tumour growth factor beta (*TGF-β*) signalling pathway, through paracrine activity [98]. Inflow of endogenous *c-kit*^*+*^ cells is also possible by thymosin β4 (*Tβ4*) administration, which in turn can lead to significant increase in survival of the transplanted cells and the vascular growth [99].

#### Umbilical cord

The MSCs derived from different compartments of the umbilical cord such as vein, arteries, Wharton’s jelly, umbilical cord lining and so forth have been observed to accumulate in damaged tissues and bolster the repair of the tissues [100]. The umbilical cord-MSCs (UC-MSCs) are said to have faster self-renewal capacity than the BM-MSCs and a lower potential of forming teratomas [101]. A very first study was performed on an animal model where the cord lining-derived MSCs combined with a vascularized omental flap ameliorated cardiac dysfunction by myocardial revascularization and attenuated remodelling [102]. Polycaprolactone nanofibres immobilized with UC-MSC-seeded fibronectin demonstrated enhanced LVEF and improved cardiac function [103]. Wharton’s jelly-derived MSCs (WJ-MSCs), obtained from embryonic epiblasts, have been identified to have properties of hESCs and adult stem cells, thereby serving as an alternative source for stem cells with significant barriers of immunorejection, tumorigenesis, teratoma formation and so forth [104, 105]. WJ-MSCs are highly specific for cardiac tissue due to their natural chemoattractive nature [105] and production of pro-angiogenic factors such as *HGF*, *VEGF*, *angiopoietin* and *TGF-β1* [106], inducing recruitment of CSCs [107]. Overexpression of N-cadherin, a cell surface gene present in UC-MSCs, leads to upregulation of *VEGF*, via the *ERK* signalling pathway [108]. Intracoronary infusion of WJ-MSCs has also been considered an alternative to BM-MSCs on the basis of their increased LVEF and decreased incidence of adverse events [109]. H_2_O_2_-preconditioned WJ-MSCs have an enhanced therapeutic effect possibly due to *IL-6* production, which leads to migration and proliferation of endothelial cells (ECs) and increased neovascularization [110]. Konstantinou et al. [111] have for the first time demonstrated the formation of cardiac polymicrotissue by differentiating hUC-MSCs using a combination of growth factors suramin and sphignosine-1-phosphate. This generated the possibility of using the polymicrotissue as a therapeutic patch over the infarct cardiac area. Similarly, umbilical-cord-derived exosome resulted in improved cardiac function by angiogenesis and their protective nature towards the myocardial tissue [112]. Also, 5-aza-induced hUC-MSCs have been observed to express *GATA-4* and *Nkx2.5* genes, and to differentiate into myocardial cells [113, 114], better than myocardial-induced fluids [115].

#### Cord blood

The haematopoietic stem progenitor cells obtained from umbilical cord blood have been studied to be very useful for clinical therapy [116–118]. However the presence of MSCs in umbilical cord blood is disputable because of the inability to obtain these cells from the gestation term cord blood [116]. On the contrary, studies suggest the presence of MSCs in fetal organs [119], with circulation in pre-term fetus blood, along with the haematopoietic precursors [120, 121]. This conflicting result has been attributed to the use of a different percentage of umbilical cord blood harvests in the two studies [116]. In the results obtained by Lee et al. [122], it is possible to extract MSCs from the cord blood that would further differentiate into mesodermal lineages. Cardiac muscles, being of mesodermal origin, can therefore also be obtained from cord blood-derived MSCs. Oxytocin exerts a promigratory effect on umbilical cord blood-derived MSCs (UCB-MSCs) [123], and the supplementation of UCB-MSCs with oxytocin results in lowered cardiac fibrosis, macrophage infiltration and restoration of *Cx-43* expression, along with a sustained ejection fraction [39]. A study established that co-transplantation of hUCB-CD34^+^ and hUC-MSCs leads to reduction in collagen deposition and improved cardiac function in MI rabbits [124].

#### Adipose tissue

The colony frequency of cells obtained from adipose tissue is higher than those of bone marrow [125] and cord blood, and these adipose tissue-derived MSCs (ASCs) can differentiate into adipocytes, chondrocytes and osteoblasts [125]. Although these cells can differentiate into vascular ECs leading to angiogenesis, along with demonstrating a paracrine effect in animal models with MI [126], cardiomyocyte differentiation is not quite feasible [127]. Under hypoxic conditions, ASCs secrete large amounts of *VEGF*, *SDF-1* and *HGF*, increasing the migration and proliferation of cardiomyocytes and reducing the apoptosis and infarct size [128]. ASCs can be isolated from the subcutaneous adipose tissue region or omental region [129]. Liver X receptor (LXR) is helpful in improving the retention and survival of the injected ASCs post MI, and when combined with ASCs leads to improvement of the cardiac function [130]. This has been studied to be possible though the toll-like receptor (*TLR*)-4/*NF-kB* and *Keap-1/Nrf-2* pathways [131]. Also, ASCs secrete various cytokines with different immunomodulatory effects which contribute a great deal in tissue regeneration [132, 133]. ASCs with overexpressed granulocyte chemotactic protein (*GCP*)-2 have resulted in enhanced angiogenic potential and survival properties [134]. Similar results were obtained for dimethyl sulfoxide-induced ASCs which differentiated into cardiomyocyte-like cells, eventually resulting in cardiac function recovery [135]. These cells have thus attracted great attention in terms of therapeutic approach towards skeletal tissue repair [132]. ASCs transplanted with hydrogel and β-galactose-caged nitric oxide donor showed improved cardiac function and enhanced cell survival [136]. ASCs embedded in scaffold containing platelet-rich fibrin are functionally superior to direct ASC transplantation, in terms of expression of *IL-10*, *Bcl-2* and *TGF-β* [137, 138]. Quite recently, another very interesting discovery made was in relation to the human adult epicardial fat surrounding the heart which served as a reservoir for mesenchymal-like progenitor cells (cardiac ATDPCs) [139]. These cells show cardiac-like phenotype despite their residence in an adipocytic environment. Also, increasing the number of cardiac ATDPCs has been shown to exert great immunosuppression [139] because of increased T-cell proliferation.

#### Skeletal muscle

Muscle-derived stem cells (MDSCs) are not restricted to myogenic or mesenchymal tissues, and can regenerate bone and muscle along with cartilage healing [140]. Satellite cells have been considered to be skeletal muscle stem cells, but they have been identified as myogenic precursors with a committed differentiation lineage that act as a reservoir of regenerative cells in case of injury [141]. Studies provide evidence for the formation of myotubes by transplantation of the satellite cell-containing myoblast into a MI model [76]. Thus, the muscle precursor cells derived from satellite cells can be considered as a viable option for regeneration of myopathic skeletal muscle [141]. MSCs obtained from skeletal muscle showed significant improvement in the LVEF of acute MI rat models, comparable with that of ASCs, but they did not transdifferentiate into cardiomyocytes or any vascular cells [142]. MDSCs have been a recent focus of study and these cells can be harvested either from orthopaedic reconstruct wastes [143] or from healthy muscle tissue biopsies [144]. The general delivery approach used for MDSCs is a tissue engineering strategy such as the use of a scaffold.

#### Placenta

The study by Vellasamy et al. substantiated the presence of MSCs in the placenta (p-SC) and suggested them as feasible regenerative medicine. Stem cells can be derived from two different parts of the placenta, namely chorionic villi and chorionic plate [145, 146]. These cells demonstrate the ability to differentiate into osteocytes and adipocytes, and show typical features of MSCs [146]. Along with their non-tumorigenic property, these cells have characteristics of both ESCs and MSCs, thereby exhibiting the capacity to differentiate into the three germ layers [147]. The major advantage of using this as a source of MSCs is that they are available in abundance as medical waste after delivery. The limitation of using p-SCs is the occurrence of high chances of impurity, since the placenta is the common medium of exchange between a mother and the baby.

#### Amnion

Amniotic mesenchymal cells (AMCs) are derived from fetal mesoderm and can be peeled off the chorionic membrane mechanically by blunt dissection [148]. These are considered a fitting cell source for cellular cardiomyoplasty by both integrating and differentiating into cardiac tissue [149]. An in-vivo study assessing the effect of AMC transplantation in a damaged myocardial tissue, in comparison with UCB-MSCs and ASCs, showed comparable results with respect to decreased infarct size, cardiomyocyte-like cell differentiation and improved cardiac function [150]. Also, these cells serve as potential curative agents due to their chemotactic characteristic [151], ample availability, lack of ethical concerns and low immune response [150]. The cardiomyogenic differentiation capacity of AMC has been shown to improve by administration of *IL-10* or progesterone [148].

#### Fibroblast

Fibroblasts are mesenchymal precursor cells that express CD34 and CD45 surface markers [152]. They migrate to the tissues via blood circulation [153], differentiating into myofibroblasts (contractile cells involved in secretion of ECM for tissue remodelling and wound healing) [152]. MSCs have been studied to promote myofibroblast congregation in the infarcted area through *TGF-β(1)-Smad2* signalling pathway [154]. An important factor discovered for myofibroblast differentiation is transient receptor potential cation channel (*TRPC6*) activity [155]. This study was conducted in vitro as well as in vivo in an experimental mice model (*TRPC6* knockout mice). The knockout mice had debilitated myofibroblast differentiation, resulting in increased ventricular dilation and reduced cardiac function [156].

Table 1 summarizes some additional information about the sources of MSCs based on frequency of production and proliferation potential in comparison with BM-MSCs, along with the techniques of administration to the intended location. The frequency of MSCs in tissues is estimated by assay of the CFU-Fs which serve as the hallmark of these cells [157]. Apart from the comparison presented in Table 1, a very interesting study by Ramkisoensing et al. investigated the differentiation potential of hMSCs derived from ESCs, fetal umbilical cord, amniotic membrane, bone marrow, adult adipose tissue and bone marrow. The results proved hESC-MSCs and fetal hMSCs to be superior to all the other MSCs co-cultured with neonatal rat cardiomyocytes, in terms of expression of most cardiac-specific genes, positive staining for α-actinin, higher basal levels of *Cx-43* and formation of capillary-like structures. Additionally, hESCs and fetal MSCs, when co-cultured with neonatal rat cardiac fibroblasts, showed no expression of α-actin and decreased* Cx-43* expression. Also unlike adult MSCs, the MSCs derived from hESCs and fetal tissue were found to differentiate into three cardiac lineages, which highlights the developmental stage of the donor tissue as a significant factor in differentiation study [158]. The MSCs derived from rat fetal heart also resulted in upregulation of anti-apoptotic, anti-fibrotic and cardiogenic growth factors when intravenously injected in a MI rat model [119]. The human fetal liver-derived MSCs have also been shown to differentiate into cardiomyocyte-like cells with a combined treatment of retinoic acid, dimethyl sulfoxide and 5-aza in high dose [159]. These cells expressed *Nkx2.5*, cardiac troponin I (*cTnT*), *Oct4* and desmin after harvesting them in the mixture.

**Table 1:** Comparison between different stem cells

	ESCs	iPSCs	HSCs	MSCs
Potency	Totipotent: zygote – morula	Pluripotent	Pluripotent	Multipotent
	Pluripotent: inner cell mass of blastocyst			
Major sources	Inner cell mass of blastocyst	Reprogramming of adult cells	Bone marrow, peripheral blood, umbilical cord blood	Bone marrow, adipose tissues, umbilical cord matrix
Cell surface markers	hESC lines: *SSEA-4, Tra 1-60, Tra 1-81* [273]	Cell surface antigenic markers expressed on ESCs, e.g. *SSEA-3* in human, *SSEA-1 *in mouse [274]	CD34 [275], CD133^+^ [276]	CD70^+^, CD90^+^, CD105^+^ [277]
	mESC lines: *NANOG, OCT4, SOX2, SSEA-1* [274]			CD34^–^ [278]
Potential clinical application in cardiac regeneration	• Yield a variety of cardiomyocyte-atrial, ventricular and sinus-nodal like cells [279] • Isolation of pure ventricular cardiomyocyte population using adenovirus vectors [280]	Generation of cardiomyocyte sheet along with endothelial cells using angiogenic. factors (VEGF) [281]	No transdifferentiation into cardiac cells in ischaemic tissues [282]	• Improves heart function • Increase in augmented angiogenesis • Reduction in fibrosis [283]
Advantages	Differentiates into three germ layers: ectoderm, mesoderm, endoderm	Produced using adult cells, hence avoids ethical issues	Proliferation and migration to site of injury	• Allogenic grafting possible without immunosuppressive agents • Limited inclination towards mutation
Limitations	• Availability of cell lines for federally funded research • Risk of producing teratomas from transplanting undifferentiating stem cells	• Generation and safe delivery of iPSC-derived cardiomyocytes is strenuous [284] • Tumour formation possible [285]	• Insufficiency in the DNA repair system caused by ageing, thereby limiting the function of HSCs [286] • Insufficient information on signalling pathway [21] • Possibility of gonadal dysfunction and infertility [287]	• Insufficient information on which MSC source to be used for the therapeutic strategy concerning a disease [19] • Route of administration is uncertain for different diseases [19]
Ethical concerns	• Involves human blastocyst • Consent for blastocyst/egg donation is required	None specifically	• Need for clinical parity • Consideration required for cure of children withess severe sickle cell disease [287]	None specifically

*ESC* embryonic stem cell, *HSC* hematopoietic stem cell, *iPSC* induced pluripotent stem cell, *MSC* mesenchymal stem cell, *VEGF* vascular endothelial growth factor

### Delivery methods of MSCs into host myocardium

Delivery of MSCs into a damaged myocardium is affected by three key factors: nature of the injury, timing of the treatment and ability of the cells to implant into the host myocardium [160]. MSCs can be delivered via several routes such as intravenous (i.v.) and i.m. injections. A study concluded improved LV function [161], improved cardiac function and higher efficiency of cell engraftment post MI in the case of i.m. injection of MSCs [162]. Also, the MSCs transplanted intramyocardially have been thought to improve myocardial lymphatic system due to their property of integrating into the lymphatic endothelium [163]. BM-MSCs when administered via intracoronary injection have been very effective in angiogenesis and improvement of cardiac function [164]. An early study for MSC delivery investigated a tissue engineering approach where two strategies are mainly applied: engineering of a stem cell-containing tissue construct or a beating cardiomyocyte-containing tissue construct [160]. For instance, to give rise to a stem cell-populated tissue construct poly(lactic–co-glycolic acid) (PLGA) [165] can be used as a scaffold and BM-MSC-derived cardiomyocyte-like cells can be used for seeded cells [166], which mimicked the structural and functional aspects of a myocardium [167, 168]. This construct was found to substantially stimulate MSC differentiation into cardiac tissue. PLGA loaded with *SDF-1α* and fabricated with coaxial electrospraying limits the contact between the protein and organic phase. When bovine serum albumin is incorporated as a carrier protein, the chemotactic effect of *SDF-1α *is enhanced and the synergistic effect leads to higher growth and proliferation of the cells [169]. Various biomaterials have been used for development of scaffold in order for it to be an ECM analogue of the host tissue. In 2014, high-density cardiac fibroblast was proposed for the development of ECM scaffolds from cardiac fibroblasts [170]. When seeded with hESC-derived MSCs, these scaffolds can be used as a delivery medium for the stem cells. In the same year Vashi et al. [171] assessed a commercial pericardial material, CarioCel, which served as a scaffold to cling onto the seeded stem cells and act as a template for formation of the new issue. A study on collagen-1 scaffold seeded with autologous MSCs demonstrated reverse modelling in rat models of chronic MI [172]. There has been limited study on the number of cells that remain localized at the site of transplantation. One such study using a hyaluronan-based scaffold for MSCs showed that although most of the cells had moved to the border leaving the scaffold, the treatment did manage to alleviate fibrosis in the area along with enhanced vascularization [173]. Hydrogel is a 3D polymeric network that swells up on exposure to water and can be of various types like collagen, fibrin, gelatin, alginate and so forth [174]. BM-MSCs with hydrogel composite have been studied to improve the cardiac functioning by preventing LV remodelling [175]. Gelatin-coated ECM dishes have also been determined as a suitable method for MSC differentiation into beating cardiomyocytes [176]. Along with preserving the structure of the matrix, this method also yields greater amounts of collagen and protein [177]. Decellularized ECMs are also used as biological scaffolds because of their advantage of being able to mimic the host ECM properties [178]. Several other ECM proteins have been identified which further lead to cardiomyocyte differentiation, protection, proliferation and angiogenesis [177]. Genipin, a natural cross-linking agent, has been utilized in various studies to fabricate biocompatible and stable hydrogels with increased stiffness and prolonged degradation. This technique does not harm the possibility of minimally invasive catheter delivery of the hydrogel [179]. Thermosensitive hydrogel has proved to be a novel method for delivering MSCs and is based on *N*-acryloxysuccinimide, *N*-isopropylacrylamide, poly(trimethylene carbonate)-hydroxyethyl methacrylate and acrylic acid [180, 181]. This hydrogel-based delivery results in higher differentiation efficiency of MSCs than co-culturing of cardiomyocytes and MSCs or chemical induction. Similarly, polytetrafluoroethylene (PTFE) and porcine small intestinal submucosa (pSIS) have been found to account for varying cell proliferation capacity of CPCs as compared with MSCs [182]. Another study determined a self-assembling polypeptide *RAD16-II*, which when mixed with cardiac marker-positive MSCs yielded a stable nanofibre scaffold, promoting cardiac regeneration at the site of tissue damage [183]. Some polymeric scaffolds lack structural integrity and thus prove to be inefficient in their delivery capacity. Thus, the use of hMSCs encapsulated in arginine–glycine–asparagine (RGD)-modified alginate microspheres helps to restore the LV function and increase the cell survival after an MI, along with enhanced angiogenesis [184]. A non-invasive cell delivery system was explored by Xu et al. where they used ultrasound-mediated bubble destruction for the delivery of drugs, genes and stem cells by upregulating *SDF-1/CXCR4* [185], and this could be used as an efficient delivery system [186]. Lee et al. developed spheroid 3D bullets from hUCB-MSCs to deliver these stem cells without the use of any cytokines [187]. The factors that seemed essential during the formation of the bullets were Ca^2+^-dependent cell–cell interaction and presence of E-cadherin as an adhesion molecule. E-cadherin activation was found to switch on the *ERK/Akt* signalling pathway required for the proliferative and paracrine activity of MSCs [187].

### Mechanisms of action of MSCs

In normal conditions of a non-injured heart, the MSCs are found to exist in low numbers, and on induction of MI these cells start proliferating rapidly for participation in wound healing, by generation of fibroblasts and myofibroblasts.

#### Homing of MSCs

The transplantation of MSCs after MI has shown that the cells infiltrate the injured tissue by trafficking through the ECM [188] and considerably repairing the cardiac function [189]. To understand the general mechanism of MSC infiltration into the damaged cardiac tissue, some studies have demonstrated the production of *HGF* by apoptotic cardiomyocytes, and not by necrotic cardiomyocytes [190]. The recruitment of MSCs has been credited to the presence of *HGF* receptor MET, which activates a wide range of signalling pathways, one of which leads to attraction of MSCs to the apoptotic cell death site [191]. This study also concluded the involvement of platelets in the migration of MSCs to the apoptotic cardiac cells through the interaction of high mobility group box-1 (*HMGB1*), which is a nuclear protein with *TLR-4* expressed on MSCs. On activation of platelet, *HMGB1/TLR-4* downregulate MET on MSCs, thereby impairing the recruitment of the cells. As a result, gene-knockout or blocking of *TLR-4* on MSCs can lead to improved infiltration of MSCs to the damaged tissue, thereby increasing the efficacy of MSC-based therapy [191].

In case of any damaged myocardium, *SDF-1α* mediates the homing of the endogenous MSCs [169]. Although the chemokine receptor *CXCR4* has not been found to be expressed in large amounts on the MSC surface, about 80–90 % of hMSCs have an intracellular storage of the receptor [192]. Following overexpression by mRNA nucleofection, the receptor stimulates Ca^2+^ signalling through its ligand *SDF-1α* [193]. SDF-1 functions as a CD34^+^ progenitor cell-recruiting agent at the site of damage in an organ [194]. However in conditions such as dilated cardiomyopathy (DCM), monocyte-chemotactic protein-1 (*MCP-1*) has been established as a homing factor of MSCs because of the presence of chemokine receptor type 2 (CCR2), a *MCP-1* receptor, on the cell surface [195]. Having said this, the further alignment of these migrated MSCs has been established and therefore additional study is required to determine whether the MSCs cause transdifferentiation, have a paracrine effect or themselves differentiate into cardiomyocytes [195]. There have been several in-vitro and in-vivo studies to understand the mechanism of MSC recruitment to the site of the damaged tissue for the reparative process to occur, along with its protective characteristic. MSCs either differentiate into beating cardiomyocytes [196], transdifferentiate or induce a paracrine effect for the regenerative process to occur.

#### Structural organization for cardiomyogenesis

Cardiac actin is the main component of thin filaments of cardiac myofibrils and sarcomere. The contraction of cardiac muscle is mediated by sarcomere [197] and troponin is an essential protein required for the cardiac muscle contractility [198] as demonstrated by a study on familial hypertrophic cardiomyopathy [199]. Beta myosin is predominantly expressed in the normal human ventricle [200]. In 2011, Wei et al. [201] conducted a study to investigate the biological characteristics of the subpopulation of MSCs that served as the therapeutic agent in heart injury and established these cells to be CPCs, due to expression of cardiac-specific markers α-actin and *cTnT* on them. The studies which used 5-aza to convert MSCs to cardiomyocytes [202], whether BM-MSCs [203] or UCB-MSCs [204], have shown the expression of all of the genes in the differentiated cardiomyocytes, such as desmin, β-myosin heavy chain, Nkx2.5 and *cTnT * A [204]. Such studies support the hypothesis that 5-aza can be useful in the reparative process of heart ventricle as well as in the amelioration of heart muscle contractility [205] (Fig. 1a). From earlier studies, *cTnT * [206] and tropomyosin [207] have been shown to play a role in Ca^2+^ regulation during contraction. Results obtained by Asumda and Chase [208] also anticipate the presence of actin in BM-MSCs, in addition to the other cardiac isoforms of troponin such as troponin I (*cTnI*), *cTnT*, troponin C (*cTnC*) and that of tropomyosin (*cTm*) which appear in the early stages of cardiomyogenic differentiation.

#### Paracrine effect

MSCs insulate the cardiac tissue from any kind of damage by reprogramming the molecular wiring of the cardiac myocytes, thereby protecting them from any hazardous compound. For instance, Rogers et al. [209] studied the therapeutic aspect of hMSCs by co-culturing them with injured myocytes from a neonatal mouse. The mouse myocytes were subjected to stress by incubating them with either toxin cytokine, *IL-1β*, or with endotoxin, lipopolysaccharide (LPS). These two compounds act as pro-inflammatory cytokines [210]. The hMSCs blocked the activation of cardiac transcription factor *NF-kB*, which is dependent on LPS, *IL-1β* [209] and *IL-6* [211, 212], thereby inhibiting the adverse effect and rendering protection to the neonatal mouse myocytes. Co-treatment of MSCs with various inflammatory factors such as *TNF-α* and *IL-1β* leads to the upregulation of vascular cell adhesion molecule-1 (*VCAM-1*) [213]. With the increase in cell adhesion ability, cardiac function was also enhanced. Several attempts have been made to protect the myocardium against ischaemia through preconditioning, which has further led to an increase in the levels of *TNF-α*, *VEGF* and *IL-8*, along with migration and recruitment of MSCs to the injured tissue [214].

In normal conditions, the cardiac fibroblasts regulate the ECM by two mechanisms: synthesis and degradation of the matrix molecules [215]. The matrix-degrading enzymes are matrix metalloproteinases (MMPs) which help the infiltrated myofibroblasts in sequential degrading of the matrix, followed by ECM synthesis. According to Wang et al. [216], MSCs affect MMP expression via the *ERK 1/2* signalling pathway, where erythropoietin may act as a paracrine factor. When MSCs of an old human, transfected with tissue inhibitor of MMP-3 (*TIMP3*) and *VEGF*, was transplanted into a rat model of MI, they showed a similar degree of angiogenic capacity to that demonstrated by young MSCs [217]. However, when young MSCs were injected into aged rat recipients, the results showed a significant decrease in scar deposition. This study thus opened up the possibility of allotransplantation of MSCs from young donors to older patients suffering from MI [218]. Neuropeptide Y (*NPY*) is a neurotransmitter present in the human central and peripheral nervous system which helps to regulate the endocrine and autonomic functions. It has been shown to promote angiogenesis with similar efficacy as fetal basic fibroblast growth factor (*fbFGF)* and *VEGF* [219]. *NPY*-induced differentiation of BM-MSCs into cardiomyocytes leads to improved angiogenesis and cardiac function along with reduced fibrosis via upregulation of *FGF-2*, cycline A2 and eukaryotic initiation factor (*EIF*)-4E genes [220]. Glycogen synthase kinase (*GSK*)-3β, when overexpressed in MSCs and injected into a coronary ligated heart, resulted in improved mortality, reduced infarct size, LV remodelling and a higher cardiomyocyte differentiation rate [221]. *GSK-3β*-MSCs also upregulated the paracrine factor *VEGF-A*, which led to increased capillary density and survival of MSCs in the tissue [221]. Similarly, genetically engineered MSCs with enhanced prostaglandin I synthase (*PGIS*) gene expression have been shown to improve cardiac function by reducing apoptosis and limiting the cardiac remodelling and increasing the VEGF-A levels, as found in a *GSK-3β* study [222]. Injection of MSCs results in activation of the *JAK*/signal transducer and activator of transcription 3 (*STAT3*) signalling pathway which has a role in the upregulation of growth factors in both diseased hearts and skeletal muscles [223]. This became evident from a study where BM-MSCs improved ventricular function in cardiomyopathic hamsters [224, 225]. The *STAT3* pathway increases the *caspase-4* level in the transplanted MSCs, and improves the post-ischaemic function by reducing pro-inflammatory and pro-apoptotic signalling in the tissue [226].

Macrophages have been another target of study to initiate the neovascularization along with MSCs [227]. Earlier studies have established that increased levels of *VEGF*, produced by *STAT3*, are the driving force behind angiogenesis in order to alleviate conditions like DCM [221, 228] and ischaemic reperfusion injury [229]. Additionally, myocardial mRNA expressions of *AT1*, *TGF-β1* and *CYP11B2* have been found to be lower in a doxorubicin-induced DCM-MSC group as compared with placebo or blank groups, where doxorubicin is administered by intraperitoneal injection in the rat model [230]. Additionally, the doxorubicin-induced injury is also possible to mitigate through BM-MSC or ASC injection [231]. The *VEGF * expression is also induced by a combined therapy of granulocyte growth factor (G-CSF) and BM-MSCs, carrying *HGF* for angiogenesis in MI rat models [232]. However, recent studies have emphasized secretion of platelet-derived growth factor (*PLGF*) factor by MSCs to promote neovascularization [233]. Hence, *PLGF* was used to check the proliferation or apoptosis of macrophages. Although no change was observed, however, a dose-dependent polarization of M1 macrophage to M2 macrophage was found to take place which released *PLGF* 50 times more than M1. This study suggested that *PLGF*, not *VEGF* secreted by MSCs, stimulates the polarization of macrophages which further secrete *PLGF* to promote neovascularization and enhance cardiac muscle repair [234] (Fig. 1b). Also, *PLGF* has been shown to directly stimulate neovascularization and hence help in cardiac repair [233].

Previous studies have underlined a significant interaction between *TGF-β1* and bone morphogenetic protein *BMP7* in the epithelial-to-mesenchymal transition for fibrosis [235, 236] (Fig. 1a). Macrophages express high *TGF-β1* [237] and MSCs express a high level of *BMP7* [238] which have a contradictory fibrogenic effect of the *TGF-β* secreted by macrophages. Another study showed improved functional recovery of the ischaemic cardiac tissue when the MSCs were co-treated with *TGF-β1* and *IL-1β*, due to an increased *VEGF* level [239].

Surgical treatment methods are mainly employed only after a patient suffers MI. This was studied in mammals for the first time based on a study of neonatal mice undergoing a 10-min surgery to induce MI [240]. This procedure leads to vascular injury [241] following which ECs synthesize cytokines, chemokines and growth factors such as *VEGF-A* [242], all of which play a protective role and stimulate the ECs along with recruitment of peripheral stem cells [242, 243]. *VEGF-A* also coordinates the differentiation of MSCs into ECs in vitro [243, 244] (Fig. 1a) and factors such as *IL-6* and *TNF-α* inhibit *VEGF-A*-induced differentiation of MSCs into ECs and subsequent capillary tube formation [245]. However, this fact has been negated in a study by Mohri et al. [246], where the authors claimed activation of the *JAK/STAT* pathway in CSCs by *IL-6* cytokines, which in turn leads to vasculogenesis of vascular endothelial precursor cells. Combined treatment of angiotensin II (*AngII*) and *VEGF-A* effectively increases the marker expression of ECs despite the presence of *IL-6* and *TNF-α*.

#### Pre-treatment and conditioning of MSCs

MSCs induced with ischaemic cardiac conditioned media showed positive reaction for *GATA-4*, *Nkx 2.5* and *MLC-2a*, suggesting cardiomyogenic differentiation of MSCs, as compared with the negligible effect by a non-ischaemic environment exerted on the MSCs [247]. Cardiomyogenic media-primed MSCs enhanced expressions of sarcomeric α-actinin and *Cx-43*, establishing themselves as better therapeutic agents than direct MSC transplantation [248].

Diazoxide is an ATP-sensitive potassium channel regulator present in the mitochondria and plays a role in suppressing apoptosis and promoting cell survival. Selected MSCs preconditioned with diazoxide resulted in an improved cell survival rate by upregulating the expression of *bFGF* and *HGF* and protecting the cells from oxidative stress injury [249].

One of the earlier studies established MSCs pre-treated with *Ang* receptor blockers (ARB) as an agent involved in improvement of cardiac function and also as a potential CSC source for cardiomyogenesis [250]. In a more recent study, it was demonstrated that *Ang II*, through an angiotensin II type-2 receptor (AT2R)-dependent mechanism, promoted the differentiation of MSCs into functional ECs [251] and upregulated the expression of *Cx-43* for gap junction formation [252] (Fig. 1c). Hence AT2R agonists and inflammatory compounds are considered key candidates for angiogenesis or vessel repair. *G9a* is a mammalian histone methyltransferase which acts as a transcriptional repressor [253]. Thus, use of BIX01294, which is a *G9a* HMT inhibitor, induced the expression of cardiac transcription factors such as *GATA-4*, *Nkx2.5* and myocardin on BM-MSCs when the cells were exposed to cardiogenic stimulating factor WNT11 [254]. Islet-1 is considered another cardiac cell marker [255], and thus progenitors with Islet-1 can differentiate into various cardiac lineages. C3H10T1/2 MSCs were used for the study of cells that differentiated into cardiomyocyte-like cells via histone acetylation [256]. These cardiomyocyte-like cells when present in the proximity of myofibres expressing collagen V show escalated integration and recovery of the infracted myocardium [257].

#### Effects of modification in MSCs

Heme oxygenase-1 (*HO-1*) when transduced into MSCs using an adenoviral vector has been shown to induce angiogenic effects [258], with enhanced anti-oxidative and anti-apoptotic capabilities [259], leading to improvement in cardiac function post MI. Human receptor activity-modifying protein 1 (*hRAMP1*) gene when overexpressed in MSCs using the same vector [260] and tagged with enhance green fluorescent protein (*EGFP*) resulted in smaller infarct size and enhanced cardiac function [261] by decreasing the *TNF-α* level, inhibiting *NF-kB* expression and enhancing the *IL-10* level [260]. *hRAMP1*-expressing MSCs are otherwise also noted to inhibit the vascular smooth muscle cell proliferation [262]. *CXCR4*-overexpressed hypoxic MSCs were also shown to enhance neovascularization, enhance EC differentiation, reduce infarct size and restore cardiac function [263]. MSCs transduced with lentiviral *CXCR4* lead to downregulation of the *caspase 3* pathways and upregulation of *pAkt* and *IGF-1α* levels [264].

An animal study used integrin-linked kinase (*ILK*)-transfected MSCs to investigate the effect on collagen synthesis and cardiac fibroblast proliferation. The study demonstrated inhibition of cardiac fibroblast proliferation and a few other factors, thereby leading to a decrease in infarct size and a reduction in fibrosis in these animals [265] along with increased cardiomyocyte proliferation [266]. Also, MSC transplantation in infarcted area has been shown to enhance the synthesis of collagen and this could be the mechanism behind attenuated ventricular remodelling post transplantation [267].

MSCs are valued for their paracrine effects in reducing inflammation [188] and promoting growth of the surrounding cells [268]. MSC injection promotes the recruitment of CPCs and helps in the improvement of myocardium [67]. Studies were performed to check the efficiency of dual cell transplantation on cardiac repair. These cells were fused to form CardioChimeras (CCs) which proved to be more efficient than single cell delivery. CPC phenotype expression dominates CCs and mediates the cardiomyogenic factors [269]. These cells also demonstrated the same phenotypic properties of commitment and high paracrine effect as those of MSCs along with increased basal expression of cardiomyogenic factors [269]. To check the effects of CCs and their parental cells, neonatal rat cardiac myocytes were incubated with them. Addition of CCs increased the expression of stromal-derived factor, a cardioprotective agent, and also acted as a ligand to *CXCR4*^*+*^ stem cells [270]. The study also showed an increase in capillary density in the area incubated with the CCs (Fig. 1b). Furthermore, the ejection fraction (fraction of blood being pumped out of the heart per heartbeat) and the anterior wall thickness of the heart also showed an improvement [269].

#### MicroRNA regulation in modified MSCs

Researchers have been investigating several other techniques to accelerate cardiac regeneration, keeping in mind the feasibility of the process. microRNAs (miRs) are approximately 22-nucleotide RNAs [271], found endogenously and involved in post-transcriptional regulation of gene expression. Some of these miRNAs are said to be cell specific or tissue specific, helping to fathom the underlying pathophysiological condition [272]. *miR-133a* is muscle specific and is proposed as a novel therapeutic target in cardiovascular disease [273]. Patients suffering from MI have been shown to have lower levels of *miR-133a* [274, 275]. *miR-133a* is known to play an important role in terminating embryonic cardiomyocyte proliferation [276], attenuating fibrosis [277] and promoting cardiac remodelling [278]. To assess its role in survival of MSCs, *miR-133a* was made to express in these cells. Researchers found that improvement in MSC survival was due to the attenuation of expression of *Apaf-1* and *caspase 9* and *3* (Fig. 1a). In contrast, depleting or blocking of *miR-133a* by its antagonist resulted in upregulation of these proteins [279]. Another significant study illustrated the overexpression of *miR-16* in cardiac-niche-induced hMSCs, when co-cultured with rat ventricular myocytes [280]. *miR-16* was found to inhibit cell proliferation, modulate the cell cycle, promote cell apoptosis and abolish tumorigenicity both in vitro and in vivo [281]. The induced cardiac niche led to dysregulation of the miRNA and increased G1 phase arrest in hMSCs, leading to their differentiation into myogenic phenotypes in the cardiac niche [280]. Similarly, *miR-499* is an embedded miRNA present within a ventricular-specific myosin heavy chain gene [282]. When overexpressed in rat BM-MSCs, *miR-499* activates the *WNT/β-catenin* signalling pathway, inducing cardiac differentiation [283]. Another mechanism of cardiac protection used by *miR-499* is calcineurin-mediated dynamin-related protein-1 (*Drp1*) activation, which prevents cardiomyocyte apoptosis [284]. *miR-34* acts as a crucial cell death regulator and its deletion or silencing reduces the age-associated cardiac cell death [285]. This occurs due to inactivation or knockdown of the stem cell factor (*SCF*), which serves as the main target of *miR-34* and thus lead to inhibition of angiogenesis [286]. *miR-23a* is also studied to regulate the *caspase 7*-induced apoptosis, involving the *TNF-α* pathway, along with a reduction in infarct size and improvement of the LV function [287].

#### Effects of treatment of MSCs on cardiac regeneration

Several compounds such as pioglitazone [288], rosuvastatin [289], *TMZ* [18, 21], gingko biloba extract 761 [290] and hydrogen sulfide [291] have been demonstrated to enhance the repair of cardiac tissue in MI models. Pioglitazone is generally used to increase the insulin sensitivity in diabetic type 2 patients. Oral intake of this drug after BM-MSC transplantation has been studied to improve cardiac function. When used in pre-treatment of MSCs, pioglitazone yielded significantly upgraded cardiac function and was even put forward as a promising CSC source for cardiomyogenesis [288] (Fig. 1c). Combined treatment of MSCs with pioglitazone showed higher levels of peroxisome proliferator-activated receptor gamma (*PPAR-γ*), which in turn led to increased *Cx-43* levels [292]. Similarly rosuvastatin, when administered with ASCs, reduced fibrosis and safeguarded the cardiac function by decreasing pro-apoptotic proteins (*Bim* and *Bam*) and increasing anti-apoptotic proteins (*Bcl-2* and *Bcl-xL*), thereby inhibiting cardiomyocyte apoptosis [289]. Hydrogen sulfide led to increased levels of phosphorylated *Erk1/2*, *Akt* and *GSK-3β*, and resulted in an increased survival rate of the transplanted MSCs, enhanced LV function and reduced infarct size [291] (Fig. 1c). Similarly, atorvastatin treatment increased the expression of *CXCR4* in MSCs, leading to enhanced migration of *SDF-1* and low levels of *IL-6* and *TNF-α* [293] (Fig. 1c). The drug also facilitated MSC survival along with improvement of LV function and decrease in the infarct size, inflammation, fibrosis and apoptosis [294]. Salvianolic acid B pre-treatment of MSCs has been found to be very effective in a rat model of MI following transplantation [295]. Improved survival of the transplanted MSCs was observed along with increase in angiogenic factors such as *VEGF*, *bFGF* and *SCF* with concomitant reduction in fibrosis and infarcted area [295] (Fig. 1c). Another study concluded that a combination of angiogenic factor genes, chemokine and stem cells could increase the angiogenesis rate and improve cardiac function [296].

MSCs have also been studied to modulate electrophysiological properties including the excitability and conduction of cardiomyocytes by two mechanisms. First, by intercellular coupling through the gap junction for reduction in instinctive activity of cardiomyocytes; and second, by increase in the conduction velocity of cardiomyocyte by paracrine signalling, via upregulation of *Cx-43* [158] and nerve growth factor [297], without any amendments in the beating frequency [298]. This therapeutic action of MSCs has been studied in a swine model, which resulted in decreased heart rate turbulence, ameliorated repolarization time and higher slope of action potential durations indicating improved cardiac functioning and reduced risk of ventricular arrhythmias [299]. In addition, MSCs have also shown to improve the contractile function and compensate for a 50 % loss of cardiomyocytes after any cardiac damage by supplementing the engineered cardiac tissues (ECTs), which serve as a 3D in-vitro model system to appraise stem cell therapies [300].

### Clinical trials using MSCs in cardiac disease performed between 2010 and 2015 and their shortcomings

There have been about 41 clinical trials (Additional file 1: Table S1) performed between 2010 and 2015 for the study of MSCs in relation to cardiac injury and repair. These trials were performed in distinct locations and were mostly found to have completed phase II, where some of them even managed to reach phase III of the study. The trials could be characterized in several ways based on their focus of study. Most of the trials focused on the injection and infusion of MSCs from different sources into the injured cardiac tissue, via different sites of injection. The second type of study compared the different kinds of MSCs (i.e. autologous and allogeneic MSCs) in context of both ischaemic as well as non-ischaemic cardiomyopathy in patients. A third type of study was performed in order to focus the safety and efficacy of these MSCs when implanted into patients, as done in a study by Da Silva and Hare [301] with the focus on the role of BM-MSCs in the treatment of chronically injured heart.

In these studies, a number of candidate cells such as neonatal and fetal cardiomyocytes, ESC-derived myocytes, skeletal myoblasts, cell types from adult BM and cardiac precursor cells have been considered. Autologous BM progenitor cells (mononuclear or MSCs) when administered myocardially resulted in improved regional contractility of the myocardial scar within 3 months of treatment [302] (ClinicalTrials.gov NCT01392625). The trial comparing the two BM preparations conducted TAC-HFT [303] and POSEIDON-DCM [304] studies to estimate the optimal cell type, delivery method, dose, mechanism of action of cell delivery and so forth. MSCs have been the main focus of these studies due to their paracrine effect, high regeneration capacity, ability to perpetuate potency and ability to avoid adverse reactions to autologous versus allogeneic transplant. A study conducted in Korea proved MSC therapy to be safe and quite efficient in terms of LVEF improvement for the treatment of acute MI [305] (ClinicalTrials.gov NCT01392105). A similar study comparing the two types of bone marrow transplants for patients with LV dysfunction due to ischaemic cardiomyopathy showed low alloimmune reactions in allogeneic MSCs and improved functional as well as structural measures when both were administered together [306] (ClinicalTrials.gov NCT01087996). Another study conducted on nine acute MI patients, following a 5-year follow-up plan to check the feasibility and safety of i.m. infection, gave a positive outcomes on MSC expansion and safety of the method and justified the possibility of placebo-controlled trials for i.m. MSC injections [307]. A similar study was conducted for i.v. allogeneic BM-MSCs in MI patients which proved to be equally efficient in improving the ejection fraction and the LV volumes [308] (Clinicaltrials.gov NCT00114452). These clinical trials have concluded the safety and feasibility of BM-MSCs, but after MI the functional recovery of the cardiac cells remains ambiguous [309]. A similar study conducted to investigate the safety and efficacy of WJ-MSCs administered via an intracoronary route demonstrated no trigger in troponin concentration as observed with BM-MSCs, indicating no coronary artery occlusion after the treatment [109] (ClinicalTrials.gov NCT01291329). Another very interesting study was conducted to observe the combined effects of stem cell implantation and mechanical circulatory support which resulted in synergistic symptomatic improvement in LV functioning [310].

Although only a few of the 41 trials have been completed and the status of some remains unknown, these trials have established various results which help bolster the upcoming clinical trials and research. On evaluating these trials, a definite trend of limitations is evident which probably played a crucial role in undermining some of the studies. Firstly, the number of patients in some of the trials was extremely lower, which could have compromised the efficacy of the study. Thus, further large-scale randomized trials are required to establish successful results. Secondly, the number of patients excluded in some studies was extremely high because of several reasons such as poor image quality after randomization. This leads to generation of unreliable and non-interpretable data. Thirdly, there is also a possibility of ignoring inter-observer and intra-observer variability in evaluating the obtained data at different intervals either due to manual error or due to unavailability of required tools such as contrast-enhanced magnetic resonance imaging (CE-MRI). Hence, Lee et al. [305] suggested the use of SPECT to minimize the inter-observer error during data analysis (ClinicalTrials.gov NCT01392105). Some trials lacked use of diverse assessment tools such as exercise tolerance, 6-min walking distance test, pulmonary function test and so forth. This could have restricted the possible varied outcomes of the trials. Another technical limitation of the trials using autologous MSCs is the inability to use them immediately since they take at least 3 weeks [311] to harvest and culture to reach an effective confluence, thereby limiting the efficiency of stem cell therapy in an acute setting. The randomized clinical trials concerning these cells need to define the period of treatment. Some experimental limitations include lack of placebo comparison groups for the study [302], which then makes the study prone to observational bias (ClinicalTrials.gov NCT01392625). Some trials evaluated only the functional aspect of the stem cell therapy, whereas others focused only on the safety and efficacy of the study, keeping the other factors constant. We anticipate more reliable outcomes if important criteria such as donor source, cell type, delivery method, dosage, cohort size and optimal time of treatment are taken into consideration.

### Challenges faced in stem cell therapy

The main challenges faced in the use of stem cells, including CSCs, for cardiovascular repair revolve around isolation of adequate stem cells, ex-vivo expansion frequency, appropriate delivery strategy and adequate differentiation and functional improvement in vivo [27]. In order to overcome the afore-mentioned challenges, MSCs have proved to be extremely efficient. Isolation of MSCs is comparatively easier; for example, the bone marrow cells can be extracted from the peripheral blood or the bone marrow itself [27]. In order to meet the increasing demand of MSCs, a microcarrier-based stirred culture system technique has been evolved for the efficient ex-vivo expansion of the stem cells, for different sources of MSCs using the various kinds of microcarriers [312]. Mesenchymal cells have thus attracted immense attention due to their therapeutic characteristics and lack of both ethical concerns and teratogenic properties [313].

#### Cell therapy precautions

Stem cell therapy has been used for the treatment of cancer, repair of damaged tissue and various degenerative diseases. The potential of such therapies was recognized long ago, leading to further developments in the field of stem-cell-based therapeutics. The success of these therapies depends on several factors such as the type of stem cell being used, its proliferative capacity and differentiation status, the route and site of administration, survival capability of the engrafted cells and so forth. On compiling these factors, a risk profile is generated that then evaluates the potential risks of the technique which can include tumour formation along with some other unwanted immune responses. As far as pluripotent cells like ESCs and induced pluripotent stem cells are concerned, they have not demonstrated any clinical risks in any of the trials. Theoretically, the high proliferation rate and unlimited self-renewal capacity of these cells constitute the risk of tumour formation. On the contrary, multipotent MSCs have not reported any major health concerns, implying the safety of MSC therapy. However, some trials have recounted serious adverse events [314], such as malignant tumour formation on transplantation of unmodified BM-MSCs in the peri-infarct area of a mouse model [315]. This calls for further investigation of the mechanisms involving MSCs. For instance, in a study conducted to observe an infarcted heart region, several calcified or ossified encapsulated structures were identified after the injection of MSCs [316]. A study on arrhythmic mechanisms established the pro-arrhythmic effects of hMSCs in neonatal rat cardiomyocytes and the pattern of the MSCs was said to be determinant of the arrhythmic severity of the myocardial tissue [317]. Another study concluded the possibility of primary cardiac sarcoma formation from MSCs, which can further develop into tumours with multi-lineage differentiation [318]. According to a study conducted by Huang et al. [319], allogeneic MSC transplantation in the myocardium exhibited a biphasic immune response of these cells, resulting in a shift from an immune-privileged state to an immunogenic phenotype after differentiation leading to characteristics such as fractional shortening and progressive ventricular dysfunction. Also, the recent investigation on electrically stimulated cardiomyocyte-like cell differentiation needs to be explored in depth [88]. Thus, evaluation of these processes tops the list of upcoming research on MSCs. Another important consideration in cell therapy is the number of passages studied in any experiment. For instance, a study based on commercially available murine MSCs showed altering expression patterns over a period of time, and this was further established by comparing the early and late passages of the model [320].

## Conclusion

**Fig. 2:**
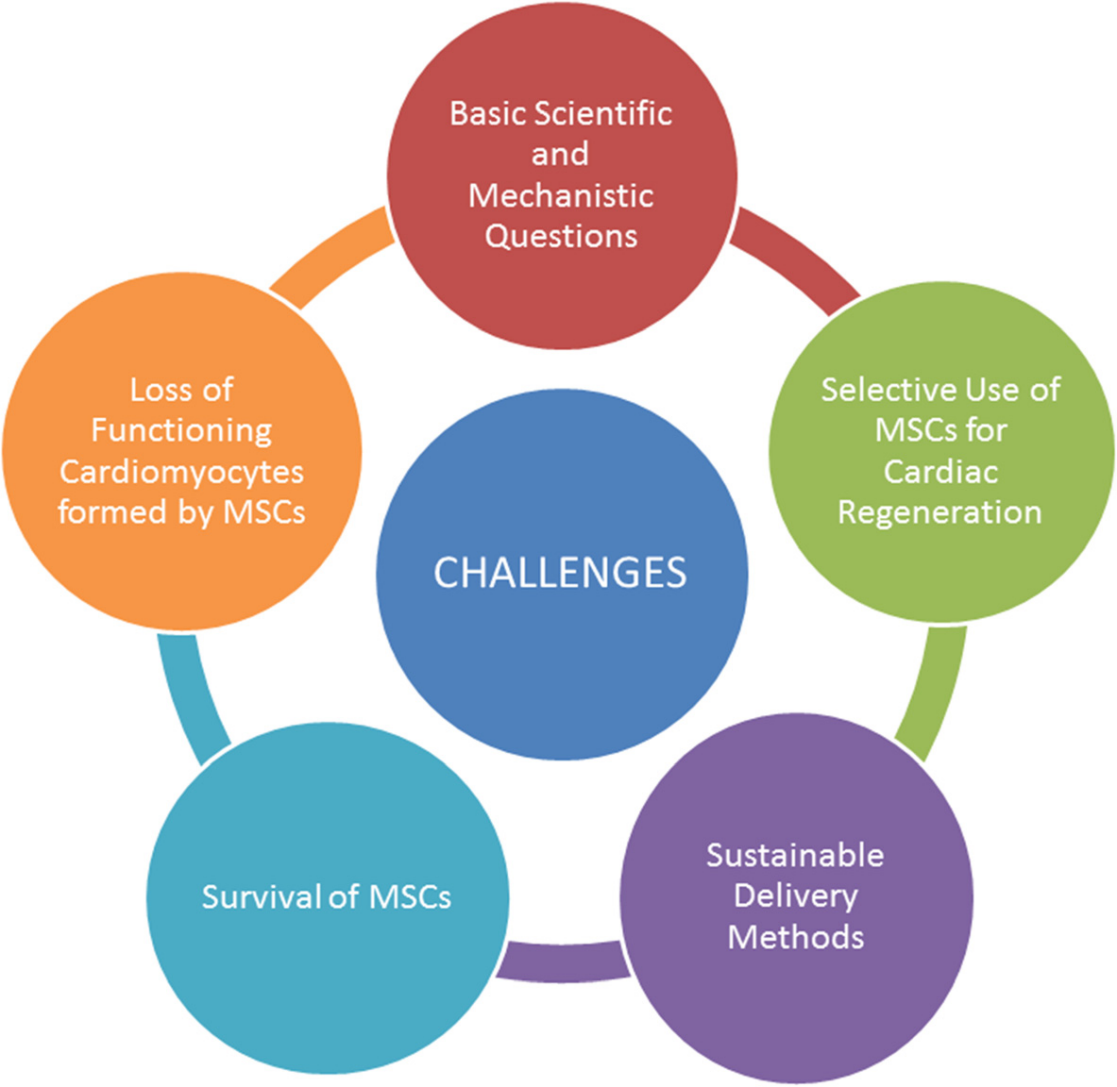
Challenges in use of MSCs for cardiac regeneration. Tumour formation in MSCs has been considered inconceivable, but there have been instances of osteosarcoma in patients infused with BM-MSCs for some other disease. Hence, in the context of MSCs in cardiac regeneration, some pathways and processes might exist that still remain unexplored. Additionally, these pathways comprise MSCs obtained from different sources, out of which only a few such as BM-MSCs have been used extensively for clinical applications, in spite of evidences of more proliferative capacity in MSCs obtained from umbilical cord, peripheral blood, etc. This limitation arises due to the lack of an efficient delivery method of MSCs to the target site. Another challenge that has seemed to come in the way of researchers is the prolonged survival of MSCs post engraftment into the host myocardium. This challenge has been overcome to a large extent by using miRNAs and CCs, but more sustainable methods need to be studied further. Studies have gained several advancements in the field of safety and efficacy of the MSC therapy, but success rates in terms of the functional regeneration of cardiac tissue for the loss of functioning cardiomyocytes after any damage remain mediocre. *MSC* mesenchymal stem cell

To evaluate the safety of MSCs in regenerative medicine, 41 clinical trials and more than 120 animal model studies have been performed since 2010 and these studies have shown MSCs to have the potential to differentiate into various mesodermal (e.g. osteoblast, adipocyte and chondrocyte) [43] and myeloid lineages [44]. The immunomodulatory characteristic of MSCs makes them a worthy competitor in the field of regenerative therapeutics. However, many pathways and underlying processes concerning MSCs still exist that remain unexplored in the field of reparative medicine (Fig. 2). Despite the therapeutic effects of MSCs, Dayan et al. [321] observed no improvements in cardiac function in a chronic ischaemic heart failure model, with no difference in the scar area, fractional shortening and so forth. A study illustrated induced and spontaneous transformation of MSCs into sarcomas in mouse, whereas in humans only induced transformation of MSCs has been observed [322]. The spontaneous transformation of hMSCs in vitro was found to be caused by the contamination of the cells by tumour cell lines [323], and studies have negated the idea of MSC transformation into tumours, even after long-term culturing of cells [324]. In contrast, in-vivo spontaneous transformation has been shown to lead to osteosarcoma genesis in patients with infused BM-MSCs for some other disease [325].

This brings us to the prospective studies in relation to the therapeutic competency of MSCs. These cells after transplantation have been shown to demonstrate paracrine effects which can prove to be of great advantage in future medical therapies (Fig. 2). Liang et al. [326] evaluated, for the first time, pigment epithelium-derived factor (*PEDF*), a paracrine factor, as a target for modifying and improving the impaired aged MSCs and thereby enhancing the cellular profile. The same is possible by overexpressing silent mating type information regulation 2 homolog 1 (*SIRT1*) in aged MSCs to restore pro-angiogenic factors, *bFGF* and so forth [327]. The regeneration process can be severely compromised by the lack of suitable MSC delivery methods to the intended site of regeneration and reduced survival of transplanted MSCs. The delivery methods for specific MSCs to the specific site of injury have yet not been established, although several delivery systems such as engineered tissue constructs and biomaterials have been explored for the same in order to gain maximum efficiency. For improving the survival of MSCs, researchers have been scrutinizing various methods which have proved beneficial under different conditions. For instance, the use of alginate-encapsulated MSCs secreting paracrine factors [328], miRNA [279] and CCs [269] has increased the survival rate of these cells. Hence, in future, the major areas of focus should involve figuring out more sustainable/evolved solutions to the afore-mentioned challenge than those under current implementation and more investigation is required in order to corroborate the efficacy of the therapies (Fig. 2).

MSCs can also be obtained from different sources in the body, but the studies in cardiac regeneration are mainly done using only a few of them. Referring to information presented in Table 2, the bone marrow has been established as one of the most promising sources of MSCs, but there have been studies indicating a higher MSC production and proliferation capacity in other parts of the body such as the umbilical cord, placenta and peripheral blood. Similarly hUC-MSCs have been found to improve motor function, reduce abnormal levels of the concerned enzymes such as lactate dehydrogenase (*LDH*), creatine kinase (*CK*), and so forth, and increase the muscle strength (ClinicalTrials.gov NCT01610440). Thus, hUC-MSCs become an important source of treatment for genetic conditions like Duchenne muscular dystrophy (DMD). A very important aspect that plays a crucial role in the treatment of cardiac disorders is the ability of any treatment strategy to compensate for the loss of the functioning cardiomyocytes [329]. Thus, one of the future challenges of cardiovascular therapies is to strategize the functional regeneration of myocardial contractility using tissue engineering, cell-based therapy or reprogramming of scar fibroblasts [330, 331].

**Table 2:** Frequency of MSC production, proliferation potential and delivery methods for therapeutic targets in different body organs, as compared with BM-MSCs

Different sources of MSCs	Frequency of production^a^	Potential of proliferation^a^	Delivery methods for regeneration	References
Bone marrow	1 in 3.4 × 10^4^ cells	–	Intravenously	[332]
Umbilical cord matrix	Low	High	Not specified	[128, 333]
Amnion	High	Low	Not specified	[334]
Placenta	High	High	Not specified	[128]
Adipose tissue	High	High	Not specified	[55, 105, 128]
Peripheral blood	High	High	Intravenously	[128, 335]
Cord blood	Low	High	Intramyocardial, intravenous, intracoronary	[103, 336, 337]

^a^In comparison with the BM-MSCs

*BM-MSC* bone marrow-derived mesenchymal stem cell, *MSC* mesenchymal stem cell

Throughout this review we came across compounds such as pioglitazone [288], rosuvastatin [289] and so forth that were studied in the initial years of the developmental era of MSCs but have not received much attention in recent years, despite the promising results obtained in cardiac therapy. There thus needs to be more research carried out on such compounds in order to not lose out on some extremely propitious therapeutic agents. Cell therapy has been adopted as a novel therapeutic strategy for treatment of cardiac disorders such as severe heart failure and CAD. Unfortunately, although these approaches have led to advancements in the field of safety and efficacy of these cell therapies, the mediocre success rates in terms of functional improvement serve as a disappointment in the field [3]. Thus we need to further investigate the sources of MSCs that can help benefit the treatment of any disorder accordingly with ‘true’ reparative potential, in order to help focus on the field of regenerative medicine.

## Abbreviations

AMC, amniotic mesenchymal cell; AngII, angiotensin II; ASC, adipose tissue-derived mesenchymal stem cell; Aza, azacytidine; BM-MSC, bone marrow-derived mesenchymal stem cell; CAD, coronary artery disease; CC, CardioChimera; CFU-F, colony-forming unit fibroblast; CPC, cardiac progenitor cell; CSC, cardiac stem cell; cTnT, Cardiac troponin T; Cx, Connexin; DCM, dilated cardiomyopathy; DM, diabetes mellitus; Drp, dynamin-related protein; EC, endothelial cell; ECM, extracellular matrix; ESC, embryonic stem cell; FGF, fibroblast growth factor; GCP, granulocyte chemotactic protein; HGF, hepatocyte growth factor; HIF, hypoxia-inducible factor; HSC, haematopoietic stem cell; i.m., intramyocardial; i.v., intravenous; LPS, lipopolysaccharide; LV, left ventricular; LVEF, left ventricular ejection fraction; MDSC, muscle-derived stem cell; MI, myocardial infarction; MMP, matrix metalloproteinase; MSC, mesenchymal stem cell; PEDF, pigment epithelium-derived factor; PLGA, poly(lactic–co-glycolic acid); PLGF, platelet-derived growth factor; PPAR-γ, peroxisome proliferator-activated receptor gamma; p-SC, placenta-derived stem cell; SDF, stromal cell-derived factor; SIRT1, silent mating type information regulation 2 homolog 1; TGF-β, tumour growth factor beta; TK, tissue kallikrein; TLR, Toll-like receptor; TMZ, trimetazidine; UCB-MSC, umbilical cord blood-derived mesenchymal stem cell; UC-MSC, umbilical cord-derived mesenchymal stem cell; VEGF, vascular endothelial growth factor; WJ-MSC, Wharton’s Jelly-derived mesenchymal stem cell.

## Additional file

Additional file 1: Table S1 Clinical trials executed during 2010–2015 which used MSCs to treat heart diseases. (DOCX 36 kb)
