# Supplementary material for: Mesenchymal stem cells in cardiac regeneration: a detailed progress report of the last 6 years (2010–2015)
Source: Stem Cell Res Ther. 2016 Jun 4;7:82. doi: 10.1186/s13287-016-0341-0 (PMC4893234; doi:10.1186/s13287-016-0341-0)
Supplement: Additional file 1: Table S1 — Clinical trials executed during 2010–2015 which used MSCs to treat heart diseases. (DOCX 36 kb) [file 13287_2016_341_MOESM1_ESM.docx]

**Additional file 1: Table S1**. List of Clinical trials executed from year 2010-2015 which used MSCs to treat heart disease

| **CLINICAL TRIAL** | **ORIGIN** | **OBJECTIVE/ RESULTS** | **PHASE** | **STUDY STATUS** | **CLINICAL TRIAL ID** |
| --- | --- | --- | --- | --- | --- |
| Administration of Mesenchymal Stem Cells in Patients With Chronic Ischemic Cardiomyopathy | University Hospital, Toulouse, France | To evaluate the safety of intramyocardial MSCs injection to repair and restore cardiac function in heart attack patients and the ones who have chronic myocardial ischemia with heart failure. | Phase I, II | Completed- Sept, 2014 | NCT01076920 |
| Effects of Intramuscular Injection of Umbilical Cord Mesenchymal Stem Cells on the Ventricular Function of Children With Idiopathic Dilated Cardiomyopathy | Qingdao University | - To study the treatment of idiopathic dilated cardiomyopathy in children using umbilical cord MSCs. - To understand the mechanism of improvement of ventricular function in the IDCM. - To gauge the safety of intramuscular UC-MSC injection. | Phase I, II | Unknown | NCT01219452 |
| A Randomized, Open-label, Multicenter Trial for the Safety and Efficacy of Intracoronary Adult Human Mesenchymal Stem Cell After Acute Myocardial Infarction | Republic of Korea  1. Yonsei University Wonju College of Medicine  2. Inha University Hospital  3. Yonsei Cardiovascular Center and Cardiovascular Research Institute | To assess the safety of intracoronary BM-MSCs in patients with acute myocardial infarction (AMI). | Phase II, III | Completed- May 2010 | NCT01392105 |
| EndocardialMesenchymal Stem Cells Implantation in Patients After Acute Myocardial Infarction | State Research Institute of Circulation Pathology, Russian Federation | Hypothesis- Percutaneous coronary intervention followed by endocardial stem cell implantation reduces scars and increases reverse remodeling in primary AMI patients. | Phase III | Ongoing | NCT01394432 |
| A Phase I/II, Randomized Pilot Study of the Comparative Safety and Efficacy of Transendocardial Injection of AutologousMesenchymal Stem Cells Versus Allogeneic Mesenchymal Stem Cells in Patients With Non-ischemic Dilated Cardiomyopathy. | University of Miami School of Medicine, Florida | Study of autologous vs. allogeneic MSCs in non-ischemic dilated cardiomyopathy patients. | Phase I, II | Ongoing | NCT01392625 |
| Mesenchymal Stromal Cell Therapy in Patients With Chronic Myocardial Ischemia (MyStromalCell Trial) | Rigshospitalet University Hospital Copenhagen, Denmark | To check the efficacy of adipose stem cells to ameliorate cardiac artery disease (CAD) in patients. | Phase II | Completed- Jan, 2014 | NCT01449032 |
| Phase 2 Study of Intracoronary Human Wharton's Jelly- Derived Mesenchymal Stem Cells (WJ-MSCs) Transfer in Patients With ST-segment Elevation Acute Myocardial Infarction (AMI) | Fu Cheng Lu 6, China | To treat patients with ST-segment elevation acute myocardial infarction using human umbilical Wharton’s jelly-derived MSCs. | Phase II | Completed- July, 2012 | NCT01291329 |
| PlasmonicPhotothermal and Stem Cell Therapy of Atherosclerosis With The Use of Gold Nanoparticles With Iron Oxide-Silica Shells Versus Stenting | - De Haar Research Foundation, Netherlands - Ural Center of Modern Nanotechnologies, Russian Federation - Ural Institute of Cardiology, Russian Federation | - Disease under study- Coronary Atherosclerosis. - Use of transcatheter micro-injection with nanoparticles to rejuvenate arteries and use of stem cells for treatment of plaque at the intended location. | Phase I | Completed- Oct, 2012 | NCT01436123 |
| Study on the Safety and Efficacy of Allogeneic Mesenchymal Stem Cell Implantation Combined With Bypass Grafting in Patients With Coronary Artery Disease and Ischemic Cardiomyopathy. | AHEPA University Hospital, Greece | To evaluate the effect of intramyocardial MSC implantation in coronary artery disease patients with ischemic cardiomyopathy. | Phase II, III | Unknown | NCT01753440 |
| Safety and Efficacy of IntramyocardialImplantation of Allogeneic Mesenchymal Stem Cells in Patients With End-stage Heart Failure Undergoing Left Ventricular Assist Device Implantation | AHEPA University Hospital, Greece | To evaluate the effect of intramyocardial allogeneic MSC implantation in coronary artery disease patients undergoing left ventricular assist device implantation. | Phase II, III | Unknown | NCT01759212 |
| Phase 1 Randomized-Double Blind Clinical Trial of Intravenous Infusion of Umbilical Cord Mesenchymal Stem CellsTransplantation in Heart Failure on Patients With Cardiopathy in Dilated Stage, of Different Etiology | Universidad de los Andes, Chile | Intravenous infusion based transplantation of umbilical cord mesenchymal cells in patients with heart failure. | Phase I, II | Completed- June, 2015 | NCT01739777 |
| A Controlled Open Label Phase II Study Assessing the Efficacy of Intracoronary Autologous Mesenchymal Stem Cells in Patients With Ischemic Dilated Cardiomyopathy | UKM Medical Centre, Malaysia | - Target of study- ischemic myocardium Malaysian patients. - To study the therapeutic capacity and differentiation potential of MSCs from CAD patients | Phase II | Ongoing | NCT01720888 |
| Phase I/II Randomized Clinical Trial to Assess the Safety and Feasibility of Transendocardial Injection of Bone Marrow Autologous Mesenchymal Stem Cells in Patients With Idiopathic Dilated Cardiomyopathy. | - Hospital General Universitario Gregorio Marañón, Spain - Hospital ClinicoUniversitario de Valladolid, Spain | To evaluate the effect of transendocardial BM-MSC implantation patients with dilated idiopathy cardiomyopathy. | Phase I, II | Ongoing | NCT01957826 |
| Ischemic Heart Failure Trial with Autologous Adipose Tissue-Derived Stromal Cells | Kanazawa University Hospital, Japan | Autologous adipose tissue derived stromal cells are administered into patients with ischemic heart failure by cardiac catherterization. |  | Ongoing | NCT01709279 |
| Preliminary Assessment of Direct Intra-Myocardial Injection of Autologous Bone Marrow-derived StromalCells on Patients Undergoing Revascularization for CAD With Depressed Left Ventricular Function | United States, Maryland | Evaluation of efficacy and safety of BM stromal stem cell by injectionsduring the heart surgery to mitigate heart muscle damage. | Phase I | Suspended | NCT01557543 |
| A Multi-center, Open-label, Comparison and a Parallel Group Study (3 Groups) Phase 3 Clinical Trial for a Comparative Evaluation With the Existing Treatments, in Order to Verify the Long-term Efficacy and Safety of the First Cell Treatment Using Hearticellgram-AMI(Autologous Human Bone Marrow Derived Mesenchymal Stem Cells) in AMI Patients, and to Observe the Efficacy of the Second Cell Treatment. | Pharmicell Co., Ltd., Seoul, Republic of Korea. | - Hearticellgram-AMI injection into AMI patients. - Compare the results with he traditional drug treatment. - Study the safety and efficacy of different doses of the hearticellgram-AMI. | Phase III | Ongoing | NCT01652209 |
| Phase I/II Study of Stem Cell Therapy in Patients With Duchenne Muscular Dystrophy | The Second Affiliated Hospital of Kunming Medical College, Yunnan, China | To study the potential of human umbilical cord mesenchymal stem cell transplantation for the Duchenne muscular dystrophy | Phase I, II | Unknown | NCT01610440 |
| Safety and Efficacy Investigation of Patients With Ischemic Cardiomyopathy by Transplantation of Umbilical Cord DerivedMesenchymal Stem Cells | The First Hospital of Hebei Medical University, Hebei, China | To evaluate the intramyocardial UC-MSC implantation for patients with ischemic cardiomyopathy | Phase I | Unknown | NCT01946048 |
| A Phase II, Randomized, Blinded, Study of the Safety and Efficacy of Transendocardial Injection of Allogeneic HumanMesenchymal Stem Cells (hMSCs) (20 Million or 100 Million Total MSCs) in Patients With Chronic Ischemic Left Ventricular Dysfunction Secondary to Myocardial Infarction. | ISCI / University of Miami, Florida, United States | Blinded study to assess the two different doses of hMSC previously studied in phase I. | Phase II | Ongoing | NCT02013674 |
| Autologous Grafting of Mesenchymal Stem Cells in Severe Refractory Ischemic Cardiomyopathy | PontifíciaUniversidadeCatólica do Paraná, Brazil | To study the intracoronary injection of MSCs in myocardial ischemic patients. | Phase I, II | Ongoing | NCT01913886 |
| A Prospective, Double Blind, Randomized, Placebo-controlled Clinical Trial of Intracoronary Infusion of Immunoselected, Bone Marrow-derived Stro3MesenchymalPrecursor Cells (MPC) in the Treatment of Patients With ST-elevation Myocardial Infarction | UMC Utrecht, Netherlands | - Double blind study, with denovo anterior MI subjects, undergoing percutaneous coronary intervention (PCI). - Intracoronary infusion of mesenchymal precursor cells in order to revascularize the left anterior descending coronary artery. | Phase II | Ongoing | NCT01781390 |
| A Phase IIa, Double-blinded, Multi-center, Randomized Study to Assess the Tolerability, and Preliminary Efficacy of a Single Intravenous Dose of Allogeneic Mesenchymal Bone Marrow Cells to Subjects With ST Segment Elevation Myocardial Infarction (STEMI) | - Mercy Gilbert and Chandler Medical Center, Arizona, US - Emory University Hospital, Georgia, US - Sanford Health Cardiovascular Institute, South Dakota, US | To evaluate the administration of human allogeneic MSCs to patients with ST segment elevation myocardial infarction (STEMI). | Phase II | Ongoing | NCT01770613 |
| H-34570: PHASE I Study: The Use of Autologous Adipose Tissue-Derived Mesenchymal Stem Cell (AdMSC) for the Improvement of Erectile and Cardiac Function in Aging Men | Baylor College of Medicine, Texas, United States | To evaluate for cardiovascular disease risk factors in men with erectile dysfunction. | Phase I | Withdrawn | NCT02107118 |
| Allogeneic Human Mesenchymal Stem Cell (hMSC) Injection in Patients With Hypoplastic Left Heart Syndrome: An Open Label Pilot Study. | - University of Maryland Medical Center, Maryland, US - Johns Hopkins Hospital, Baltimore, US | To study intramyocaridal allogeneic MSC injection during the Bi-Directional Cavopulmonary Anastomosis (BDCPA) surgery for pediatric patients with hypoplastic left heart syndrome (HLHS). | Phase I | Ongoing | NCT02398604 |
| Prospective, Randomized, Multi-center Phase 2 Clinical Trial of Allogeneic Bone Marrow-derived Human Mesenchymal Stem Cells for the Treatment of Acute Respiratory Distress Syndrome | - University of California San Francisco, US - Stanford University, US | To study the efficacy and safety of a single dose of Allogeneic Bone Marrow-derived hMSCin patients with Acute Respiratory Distress Syndrome (ARDS). | Phase II | Ongoing | NCT02097641 |
| The Efficacy and Safety Assessment of Human Umbilical Cord Stroma-derived Multipotent Stromal Cells in Myocardial Infarction; a Phase 1/2 Clinical Trial (HUC- HEART Study) | Alp Can, Turkey | To evaluate the safety and efficacy of allogeneic UC stroma- derived MSC for patients with myocardial infarction. | Phase I, II | Ongoing | NCT02323477 |
| Allogeneic Adipose Tissue-derived Stromal/ Stem Cell Therapy in Patients With Ischemic Heart Disease and Heart Failure - a Safety Study | Department of Cardiology, The Heart Centre, Denmark | Clinical safety trial with adipose-derived mesenchymal stem cells in heart failure patients | Phase I | Completed- Nov, 2015 | NCT02387723 |
| A Double-blind, Randomized, Sham-procedure-controlled, Parallel-group Efficacy and Safety Study of Allogeneic Mesenchymal Stem Precursor Cells (CEP-41750) in Patients With Chronic Heart Failure Due to Left Ventricular Systolic Dysfunction of Either Ischemic or Nonischemic Etiology | United States | To identify the efficacy of transendocardial allogeneic mesenchymal precursor cells in treatment of LV systolic dysfunctional heart failure. | Phase III | Ongoing | NCT02032004 |
| A Phase II, Randomized, Placebo-Controlled Study of the Safety, Feasibility, and Efficacy of Autologous Mesenchymal Stem Cells and C-kit+ Cardiac Stem Cells, Alone or in Combination, Administered Transendocardially in Subjects With Ischemic Heart Failure | - Stanford University School of Medicine, California, US - University of Florida-Department of Medicine, Florida, US - University of Miami-Interdisciplinary Stem Cell Institute, Florida, US - Indiana Center for Vascular Biology and Medicine, Indiana, US - University of Louisville, Kentucky, US - Minneapolis Heart Institute Foundation, Minnesota, US - Texas Heart Institute, Texas, US | To evaluate the safety and efficacy of transendocardial injection of autologous BM-MSC and c-kit cardiac stem cells, individually and in combination, in patients with ischemic cardiomyopathy. | Phase II | Ongoing | NCT02501811 |
| Mesenchymal Stem Cells Administration in the Treatment of Coronary Graft Disease in Heart Transplant Patients | Département de cardiologie du Pr Michel KOMADJA, France | To assess the intramyocardial BM-MSC injection in patients with cardiac transplant with severe coronary vasculopathy. | Phase I, II | Ongoing | NCT02472002 |
| Safety and Efficacy of Human Umbilical-Cord-derived Mesenchymal Stem Cell Transplantation in Ischemic Cardiomyopathy | Department of cardiology,Affiliated Hospital to Academy of Military Medical Sciences, Beijing, China | A single blind, randomized study to assess the UC-MSC injection in chronic heart ischemia patients. | Phase I, II | Ongoing | NCT02439541 |
| Intravenous Administration of Allogeneic Bone Marrow DerivedMultipotentMesenchymal Stromal Cells (MSCs) in Patients With Recent Onset Anthracycline-Associated Cardiomyopathy | University of Texas MD Anderson Cancer Center, Texas, US | To evaluate the effects of addition of mesenchymal stem cells to the drugs treating anthracycline-induced heart failures. | Phase I | Ongoing | NCT02408432 |
| A Phase I, First-in-Human, Multicenter, Randomized, Double-Blinded, Placebo-Controlled Study of the Safety and Efficacy of Allogeneic Mesenchymal Stem Cells in Cancer Survivors With Anthracycline-Induced Cardiomyopathy | - Stanford University School of Medicine, California, US - University of Florida-Department of Medicine, Florida, US - Indiana Center for Vascular Biology and Medicine, Indiana - University of Louisville, Kentucky - Minneapolis Heart Institute Foundation, Minnesota - Texas Heart Institute, Texas, US | - To study the effect of allogeneic MSC injection to cancer survivors suffering from anthracyclin- induced cardiomyopathy, along with LV dysfunction. - To acquire therapeutic evidence of the treatment. | Phase I | Ongoing | NCT02509156 |
| Pilot Study of Investigation of Bone Marrow-derived Mesenchymal Stem Cells (MSC) Administration in Weaning From Left Ventricular Assist Device | Cardiology Department of Rangueil Hospital - Rangueil Hospital, France | Use of MSC therapy and left ventricular assist devices to wean the chronic myocardial ischemia patients after transplantation. | Phase I | Ongoing | NCT02460770 |
| A Phase IIa Randomized Study to Assess the Safety, Tolerability, and Preliminary Efficacy of a Single Intravenous Dose of Ischemia-tolerant Allogeneic Mesenchymal Bone Marrow Cells to Subjects With Non-ischemic Heart Failure | - Emory University Hospital, Georgia, US - Northwestern University Centers for Heart Failure Therapy, Illinois, US - Stony Brook Heart Institute, New York, US - Hospital of the University of Pennsylvania, Heart Failure and Transplant Program, Pennsylvania, US | Study of patients with non-ischemic heart failure, intravenously injected with ischemia-tolerant allogeneic mesenchymal bone marrow cells. | Phase II | Ongoing | NCT02467387 |
| A Phase I/II, Randomized, Placebo-Controlled Study of the Safety and Efficacy of Transendocardial Injection of Autologous Human Cells (Mesenchymal or the Combination of MSC and Cardiac Stem Cells) in Patients With Chronic Ischemic Left Ventricular Dysfunction and Heart Failure Secondary to Myocardial Infarction. | ISCI / University of Miami, Florida, US | Pilot study to test the composition of cells introduced using a catheter system and to obtain a safety data. | Phase I, II | Ongoing | NCT02503280 |
| Safety and Exploratory Efficacy Study of UCMSCs in Patients With Ischemic Heart Disease (SEESUPIHD) | China | To evaluate the safety and efficacy of UC-MSC for patients with ischemic heart diseases. | Phase I, II | Ongoing | NCT02568956 |
| Randomised, Double-blind, Placebo-controlled, Intracoronary or Intravenous Infusion Human Wharton' Jelly-derivedMesenchymal Stem Cells in Patients With Ischemic Cardiomyopathy | Beijing | To assess the intravenous or intracoronary Wharton’s Jelly- derived MSC injection in patients with myocardial ischemia, followed by ischemic cardiomyopathy. | Phase II | Ongoing | NCT02368587 |
| Effect of IntramyocardialMesencymal Stem Cells Injection in Patients With Chronic Ischemic Cardiomyopathy and Left Ventricular Dysfunction Guide by NogaStar XP System Catheter. | Cardiology Department of Rangueil Hospital - Rangueil Hospital, France | Multicenter, randomized, double-blind, placebo-controlled study to focus on the functional improvement of the MESAMI program by measuring peak VO2, between the placebo group and the cell therapy group. | Phase II | Ongoing | NCT02462330 |
| The Safety and Efficacy Assessment of Human Umbilical Cord-derived Mesenchymal Stem Cells (hUC-MSCs) With Injectable Collagen Scaffold Transplantation for Chronic Ischemic Cardiomyopathy | The Affiliated Nanjing Drum Tower Hospital of Nanjing University Medical School, Jiangsu, China | To study the efficacy and safety of transplanted human UC-MSC in association with injectable collagen scaffold, in chronic ischemic cardiomyopathic patients. | Phase I, II | Ongoing | NCT02635464 |
| Therapy of Preconditioned Autologous BMMSCs for Patients With Ischemic Heart Disease (TPAABPIHD) | China | To study the efficacy of preconditioned BM-MSC injection for ischemic heart disease patients. | Phase I, II | Ongoing | NCT02504437 |
